# Clinical characteristics and genotype–phenotype correlation analysis in 279 patients with 5α-reductase type 2 deficiency

**DOI:** 10.3389/fendo.2026.1919643

**Published:** 2026-09-25

**Authors:** Kun Wang, Bingbing Shi, Lili Huang, Lin Qiao, Lin Liao, Tian Tang, Jie Bai

**Affiliations:** 1 Department of Endocrinology and Metabology, Liaocheng People’s Hospital, Liaocheng, China; 2 Department of Endocrinology and Metabology, Liaocheng People’s Hospital Affiliated to Shandong First Medical University, Liaocheng, China; 3 Department of Endocrinology and Metabology, The First Affiliated Hospital of Shandong First Medical University & Shandong Provincial Qianfoshan Hospital, Jinan, China

**Keywords:** 5α-reductase type 2 deficiency, differences/disorders of sex development, genotype-phenotype correlation, geographic heterogeneity, SRD5A2

## Abstract

**Objective:**

5α-Reductase type 2 deficiency (5α-RD2) is an autosomal recessive differences/disorders of sex development caused by *SRD5A2* gene mutations, characterized by impaired testosterone-to-dihydrotestosterone conversion and highly heterogeneous clinical phenotypes. The genotype-phenotype correlation of 5α-RD2 remains incompletely understood, and large-scale multicenter evidence is lacking for clinical management.

**Methods:**

We systematically searched PubMed, Embase, CNKI, Wanfang, and Chinese Medical Journals databases from inception to December 2025. A total of 279 genetically confirmed patients with 5α-RD2 from 36 countries (64.77% from Asia) were included. Demographic, clinical, biochemical, genetic, and prognostic data were extracted and analyzed using SPSS 25.0.

**Results:**

Seventy-eight pathogenic *SRD5A2* mutations were identified, with missense mutations being the most common (64.10%) and p.R246Q the most prevalent variant. The mutation spectrum showed significant geographic heterogeneity. Consanguinity was strongly associated with homozygous mutations (*P* < 0.001), and patients with homozygous mutations had significantly more severe undermasculinization than compound heterozygotes (*P* = 0.011). p.R246Q, p.G203S, and p.R246W were significantly associated with a higher likelihood of hypospadias; p.R227Q was significantly associated with a lower likelihood of cryptorchidism and scrotal abnormalities. Frameshift mutations were associated with impaired pubertal development (*P* = 0.013). Homozygous carriers were diagnosed significantly later than heterozygotes (*P* = 0.029), and serum FSH levels positively correlated with phenotypic severity (*P* = 0.021).

**Conclusion:**

We verified that the genotype–phenotype correspondence is non-linear and multifactorial, jointly modulated by mutation subtype, residual enzyme activity, endocrine axis status, geographical disparities, consanguinity, and regional medical resource availability. These findings provide more reliable evidence for clinical diagnosis, treatment, and prognosis assessment.

## 1 Introduction

5α-Reductase type 2 deficiency is an autosomal recessive differences/disorders of sex development (DSD) affecting 46,XY individuals, caused by pathogenic variants in the *SRD5A2* gene encoding the steroid 5α-reductase type 2 isoenzyme (1–3). This enzyme catalyzes the conversion of testosterone (T) to dihydrotestosterone (DHT) (4), which is essential for normal virilization of the external genitalia during fetal development (1). In affected patients, impaired DHT production results in incomplete or absent masculinization of the external genitalia despite normal testicular development and T secretion (5).

The clinical spectrum of 5α-RD2 is remarkably wide, ranging from completely female-appearing external genitalia at birth to isolated micropenis or hypospadias (6–8). A distinctive feature is spontaneous virilization at puberty, including voice deepening and phallic enlargement (4, 9), likely due to alternative DHT synthesis via the type 1 isoenzyme (3). Epidemiologically, the disease shows marked geographic clustering in regions with high consanguinity (e.g., the Dominican Republic, Turkey, and Papua New Guinea) (1, 2, 10, 11). Its true incidence is likely underestimated, as diagnosis remains challenging in neonates and prepubertal children.

The molecular basis has been extensively studied: the *SRD5A2* gene comprises five exons encoding a 254-amino acid protein, with over 100 pathogenic mutations identified to date, among which missense mutations predominate and exons 1 and 4 are mutational hotspots (12, 13).

Despite substantial progress, several critical issues remain unresolved. The genotype–phenotype correlation is highly controversial: although some structural studies have linked specific mutation categories (e.g., NADPH-binding site mutations) to milder phenotypes (14), other reports show no clear association, and functional assays often fail to predict clinical severity (15). Diagnosis remains challenging, as serum DHT and T/DHT ratios after human chorionic gonadotropin (hCG) stimulation are often equivocal, making *SRD5A2* mutational analysis the gold standard for confirmation (16). Furthermore, initial sex assignment is influenced by cultural and social factors rather than virilization degree alone, and long-term outcomes regarding psychosexual development, fertility, and quality of life have not been systematically evaluated (17, 18).

Given these unresolved controversies, further large-scale studies are needed to comprehensively clarify the genetic and clinical landscape of 5α-RD2. In the present study, we conducted a systematic review and pooled analysis of 279 genetically confirmed patients from multiple centers worldwide. We aimed to: (1) delineate the global mutation distribution patterns; (2) characterize the full phenotypic spectrum; and (3) investigate the potential genotype–phenotype correlation, with the goal of providing robust evidence to guide clinical diagnosis, treatment, and genetic counseling for affected individuals and their families.

## 2 Methods

### 2.1 Data sources and study subjects

We systematically searched PubMed, Embase, China National Knowledge Infrastructure (CNKI), Wanfang, and Chinese Medical Journals from inception to December 2025 without language restrictions, using “*SRD5A2*” as the primary search term. We performed supplemental tracing during data extraction: whenever the main text of an included article mentioned additional patients diagnosed with 5α-RD2 via genetic testing, we identified and sourced the original publications to extract the relevant data.

Included studies met: (1) definitive diagnosis by *SRD5A2* mutational analysis; and (2) relatively complete clinical and genetic data for the proband. Extracted variables included demographics, age at diagnosis, rearing gender, consanguinity, clinical features, laboratory findings (with hCG stimulation data extracted when available), mutation details, and therapeutic outcomes. Individual-level genetic data for all included patients are provided in **Supplementary Table 1**.

### 2.2 Phenotype assessment

External genital masculinization was assessed using the Sinnecker scale (19) and External Masculinization Score (EMS) (20). Since these scores were rarely reported directly, two independent reviewers retrospectively calculated them from detailed narrative descriptions using standardized templates. Disagreements were resolved by consensus. Cases with insufficient descriptions were recorded as missing (**Supplementary Material 1**).

### 2.3 Statistical analysis

Analyses used SPSS 25.0. Categorical data were compared using χ² or Fisher’s exact tests. Continuous data were tested for normality; normally distributed data were compared using t-test/ANOVA, and non-normally distributed data using Kruskal-Wallis H or Mann-Whitney U tests. Correlations used Pearson or Spearman methods.

Because included studies used heterogeneous hCG protocols (varying dosages/frequencies/durations), post-hCG T, DHT, and T/DHT data were not included in core statistical analyses and are presented descriptively. To harmonize data, all hormone concentrations were converted to molar units (nmol/L) before calculating ratios, and analyses were stratified by age (infancy/childhood/adulthood). Non-parametric tests were used to minimize assay-related variability.

All analyses used complete-case analysis without imputation; effective sample sizes are reported for each analysis. Consanguinity was analyzed as a dichotomous variable against zygosity, mutation type, and Sinnecker grade using χ² tests or ordinal/multinomial logistic regression as appropriate. A two-sided *P* < 0.05 was considered significant.

## 3 Results

### 3.1 Basic characteristics of included subjects

A total of 279 genetically confirmed patients with 5α-RD2 were included. Of these, 264 cases (94.62%) had documented geographic origin, distributed across 36 countries on four continents, with the majority from Asia (64.77%) (**Figure 1**; **Supplementary Table 2**). Age at diagnosis ranged from 4 days to 57 years: 35.16% (77/219) diagnosed in infancy (0–3 years), 26.48% (58/219) in childhood (4–12 years), and 38.36% (84/219) in adulthood (≥13 years). Four patients were initially misdiagnosed with Androgen Insensitivity Syndrome (AIS). Among 175 patients with available parental marital data, 40.57% (71/175) were from consanguineous unions and 58.28% (102/175) from non-consanguineous unions. Family history related to DSD was documented in 60 patients: a positive family history was confirmed in 41.67% (25/60), negative in 50.00% (30/60), and unknown in 8.33% (5/60). A complete list of individual participant genetic results, organized by study, is available in **Supplementary Table 1**.

**Figure 1:**
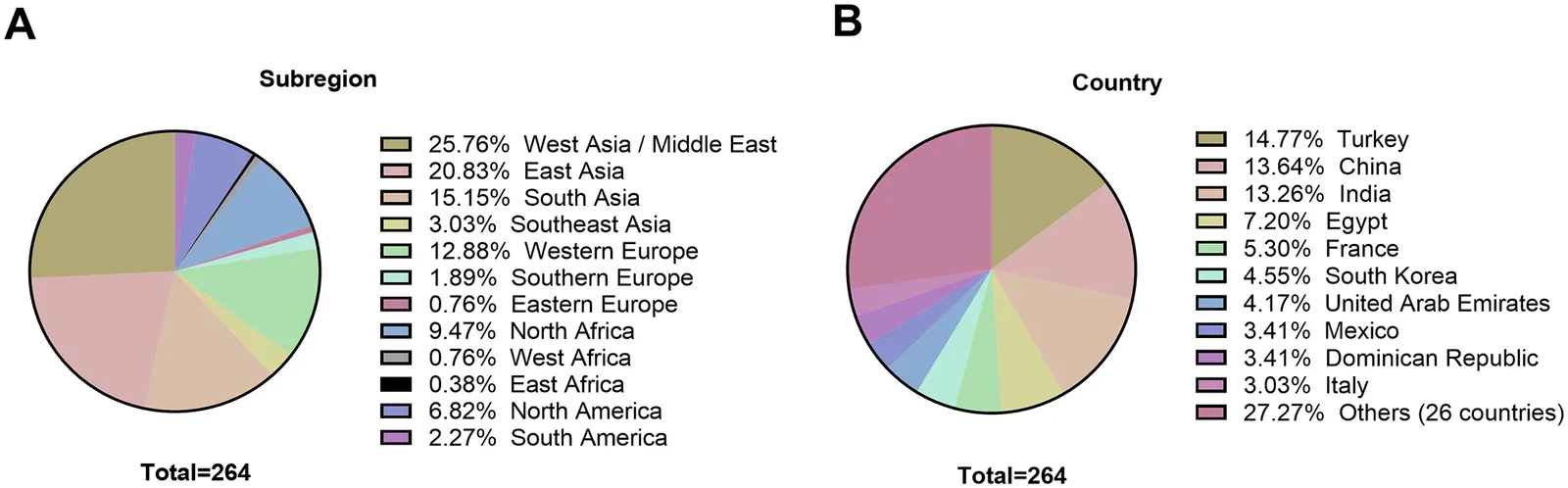
Geographic distribution of 264 patients with 5α-RD2 by subregion and country. Pie charts show the distribution of 264 patients by subregion **(A)** and country **(B)**. Percentages are based on patients with documented geographic origin (n = 264); 15 patients lacking geographic data were excluded. Countries with fewer than 8 cases (n < 3.03%) are grouped as “Others” (26 countries, accounting for 27.27% of the total). The subregional classification was based on the geographic regions defined by the World Health Organization (WHO).

### 3.2 Clinical characteristics

#### 3.2.1 External genital phenotype

External genital masculinization was assessed using the Sinnecker scale (available for 250/279, 89.6%) and EMS (available for 214/279, 76.7%). Based on the Sinnecker classification, 28.34% (70/247) presented with predominantly male external genitalia, 24.70% (61/247) with ambiguous genitalia, and 46.96% (116/247) with predominantly female genitalia. EMS scores ranged from 0.0 to 11.0.

#### 3.2.2 Hormonal profiles

Baseline serum T, DHT and T/DHT ratios were available for 113 patients (40.5%), and post-hCG stimulation data for 97 patients (34.8%). In pediatric and adult patients, baseline T/DHT ratios ranged from 2.5 to 144.9, with 77.08% (37/48) >15. Post-hCG T/DHT ratios ranged from 1.05 to 187.7, with 66.67% (50/75) >15. In 33 infants, baseline T/DHT ratios ranged from 0.8 to 45.4, with 42.42% (14/33) >10.

#### 3.2.3 Pubertal development and growth

Tanner staging was documented in 23 patients (8.2%): Stage I in 4.35% (1/23), Stage II in 17.39% (4/23), Stage III in 21.74% (5/23), Stage IV in 21.74% (5/23), and Stage V in 34.78% (8/23).

Height SDS was available for 19 patients and weight SDS for 13 patients. Neither height SDS nor weight SDS differed significantly from age-matched healthy male norms (*P* = 0.300 and *P* = 0.638, respectively). A significant negative correlation was observed between age and height SDS (r_s_=-0.488, *P* = 0.034) (**Supplementary Table 3**).

Breast development was recorded in 32 patients: 5 showed breast tissue changes (1 after testosterone replacement therapy and 4 spontaneous, including 1 incomplete and 2 mild bilateral), while 27 showed no evidence of breast development. Bone age, available for 3 patients (one each in infancy, childhood, and adulthood), matched chronological age in all cases.

#### 3.2.4 Interventions and outcomes

Surgical interventions were performed in 78 patients. Among 27 male-reared patients, hypospadias repair was the most common procedure (51.85%, 14/27), followed by fixation procedures (29.63%, 8/27), plasty (11.11%, 3/27), chordee correction (7.41%, 2/27), and scrotoplasty (3.70%, 1/27). Among 49 female-reared patients, all underwent gonadectomy; 18 also received concurrent reconstructive surgeries. Gonadectomy was performed in infancy in 48.15% (13/27), childhood in 40.74% (11/27), and adulthood in 11.11% (3/27). Pathological examination (4 cases) revealed no malignancy in 3 cases and one solid tumor with necrosis (21).

Pharmacological treatment was documented in 39 patients: androgen therapy in 25 male-reared patients and estrogen therapy in 15 female-reared patients. Among 25 patients receiving androgen therapy, penile stretched length increased by 0.5–3.0 cm from baseline (9.09%–420% relative increase). Treatment duration showed a moderate positive correlation trend with penile growth (n=14, r=0.410, *P* = 0.145; n=15, r_s_=0.376, *P* = 0.168).

#### 3.2.5 Gender assignment and identity

Initial rearing gender was documented for 241 patients: 31.54% (76/241) male, 68.05% (164/241) female, and one with gender fluidity. Social gender change (female to male) occurred in 44 cases. Gender identity disorder was documented in 10 patients; among nine initially reared as female, five identified as male, five underwent social gender transition, two had gender dysphoria, and two had undifferentiated gender identity.

#### 3.2.6 Reproductive outcomes

Fertility data were documented in 16 patients (5.7%). Eleven patients had children: six through assisted reproductive technology (ART), three through natural conception, and two with unclear mode. Semen analysis (available in 13 patients) showed oligozoospermia in 10 cases, azoospermia in 1, and normal sperm concentration in 2.

### 3.3 *SRD5A2* gene mutation analysis

#### 3.3.1 Mutation types and distribution

Seventy-eight pathogenic mutations were identified: 50 missense (64.10%), 12 deletions (15.38%), 6 nonsense (7.69%), 6 splice-site (7.69%), 2 read-through (2.56%), and 2 large deletions. Exons 1 and 4 were mutation hotspots (34.62% and 25.64%, respectively). The most common mutation was p.R246Q (44/344, 12.79%), followed by p.G203S (21/344, 6.10%) and p.R227Q (20/344, 5.81%).

In terms of zygosity, 187 cases (70.04%) were homozygous and 80 (29.96%) were compound heterozygous. Among homozygotes, p.R246Q was most frequent (26/187, 13.90%); among compound heterozygotes, p.R246Q/p.G203S predominated (6/80, 7.50%).

Significant geographic heterogeneity was observed in mutation distribution. p.R246Q, p.G203S, p.R227Q, p.L55Q, and p.Q6X were enriched in Asian populations (all *P* < 0.05); p.G34R was Africa-specific (OR = 61.750, *P* < 0.001); and p.H231R and p.R246W characterized European and American populations, respectively (both *P* < 0.001) (**Figure 2**; **Supplementary Table 4**).

**Figure 2:**
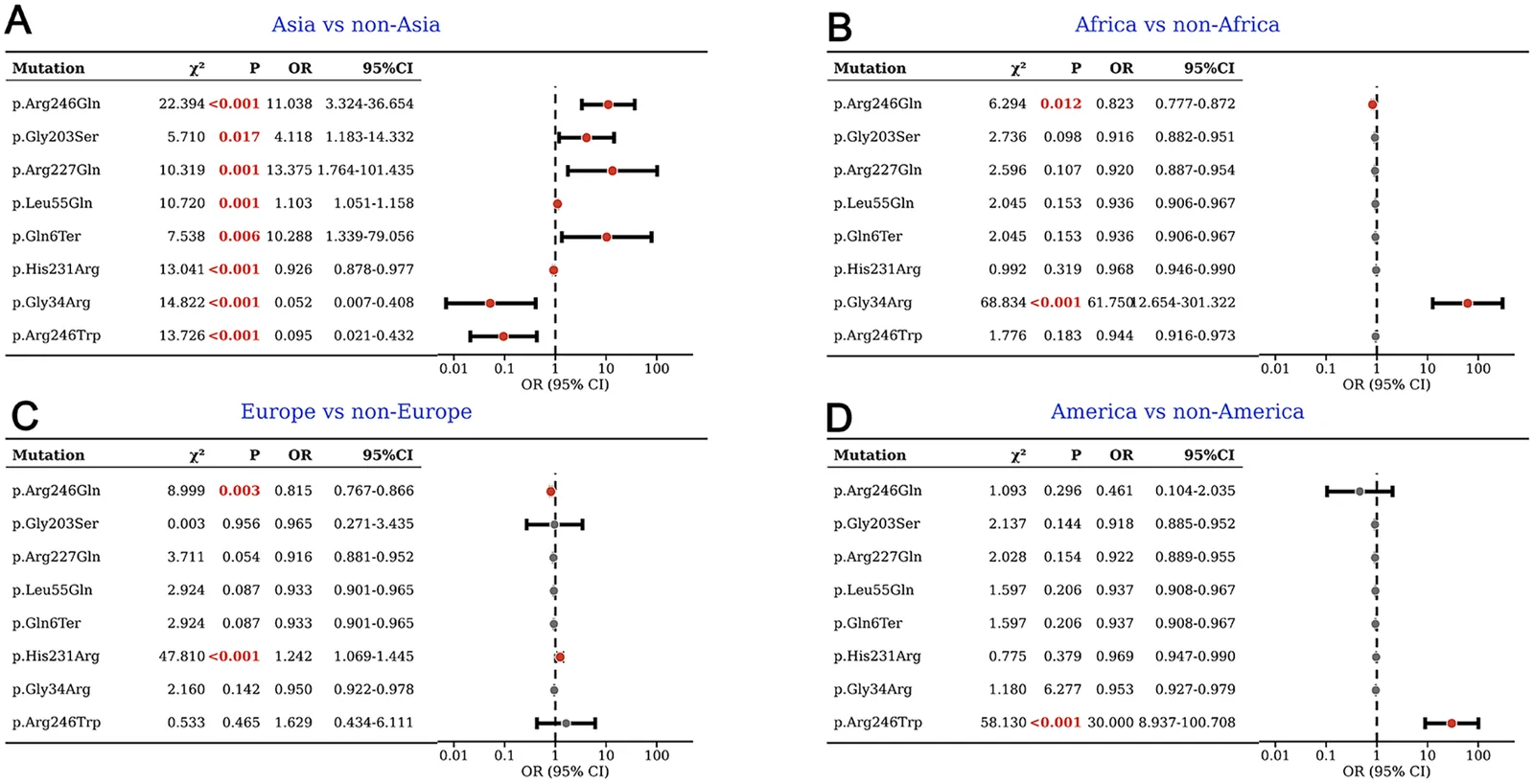
Geographic heterogeneity of *SRD5A2* mutation distribution across continents. Forest plots show the regional enrichment of recurrent *SRD5A2* variants in Asia **(A)**, Africa **(B)**, Europe **(C)**, and America **(D)** compared with non-corresponding regions. Significant associations are highlighted in red.

#### 3.3.2 Residual enzymatic activity by mutation type

Splice-site, nonsense, and certain missense mutations resulted in complete loss of enzyme activity (0% residual); frameshift and some missense mutations led to markedly reduced activity (<10%); whereas p.V89L and p.G203S showed mild functional impairment (40%–70% residual) (**Table 1**).

**Table 1:** Residual enzymatic activity and molecular mechanisms of different *SRD5A2* gene mutations.

Mutation types	Mutation Names	Molecular mechanism	Residual enzyme activity
Splice Mutation	c.755 + 1G>T	Directly causes functional enzyme inactivation (22);	0%
	c.755 + 1G>T/p.R246Q	Results in approximately 97% loss of enzyme function (23);	3%
Nonsense Mutation	p.Q6X	Production of protein truncation, complete loss, and NADPH-binding site (NADPH, nicotinamide adenine dinucleotide phosphate) defects (21, 24–27);	0%
	p.Q71X	Leads to protein truncation (27);	—
Frameshift Mutation	c.122_123delAG	Premature stop codon occurs, producing non-functional truncated protein (28);	—
	c.188_189delTG	Premature stop codon occurs, producing non-functional truncated protein (29);	—
	c.453delC	Premature stop codon occurs, producing non-functional truncated protein (30);	—
	c.418delT	Premature stop codon occurs, producing non-functional truncated protein (31);	—
	c.655delT	The formation of proteins carrying abnormally elongated termination signals (32);	1%
Missense mutation	p.G115D	Complete loss of enzyme activity (4, 33, 34);	0%
	p.R246W	Significantly enhanced affinity between the enzyme and NADPH (Nicotinamide Adenine Dinucleotide Phosphate, Reduced form) and shortened protein half-life were observed (23, 33);	<5%
	p.R246Q	Direct impairment of NADPH-binding capacity, alteration of the enzyme’s optimal pH (23, 35–38);	—
	p.R227Q	Altered substrate and NADPH (Nicotinamide Adenine Dinucleotide Phosphate, Reduced form) binding, with a significantly decreased catalytic rate (39, 40);	3.2%
	p.G183S	Enzyme–NADPH affinity (41);	10%
	p.E200K	Affects the binding capacity between the enzyme and its cofactors (42);	—
	p.I159R	Alters the secondary structure of the protein (43);	—
	p.Y242D	Directly acts on the binding region of the enzyme (44);	—
	p.H232R	Affects critical domains, resulting in enzyme inactivation (45);	0%
	p.G196S	Significantly increased the Km value of NADPH (Nicotinamide Adenine Dinucleotide Phosphate, reduced form) and altered the optimal pH of the enzyme (4);	5.7%
	p.V89L	Residual enzyme activity is approximately 70% (46, 47);	70%
	p.G203S	Residual enzyme activity is approximately 40% (14, 48);	40%
Special types	p.M1I	Translation start site shifting and affinity between NADPH and the enzyme (49);	—
	p.E197D accompanied by UPD	Introduction of EcoR V restriction enzyme cleavage site (50–53);	—
	p.X255Q	Results in the replacement of the stop codon by a glutamine codon, leading to a protein extended by 28 amino acids (28);	—

NADPH stands for nicotinamide adenine dinucleotide phosphate; UPD refers to uniparental disomy; Km denotes the Michaelis constant. The symbol “—” indicates that there is no reported data regarding residual enzyme activity in the literature. Residual enzyme activity is calculated based on 100% activity of the normal wild-type enzyme.

### 3.4 Genotype–phenotype correlations

#### 3.4.1 External genital phenotype

Mann-Whitney U test revealed significantly more severe undermasculinization in homozygous compared to compound heterozygous patients (Z=-2.532, *P* = 0.011; median rank: 106.51 vs 85.32) (**Supplementary Table 5**).

Kruskal-Wallis H test showed significant overall differences in phenotypic severity among mutation types (χ²=9.942, df=4, *P* = 0.041). *Post-hoc* pairwise comparisons with Bonferroni correction showed no individual comparison reached statistical significance, though the difference between missense and splice-site mutations approached significance (adjusted *P* = 0.084) (**Supplementary Table 6**).

Pearson χ² tests examining specific mutation–phenotype associations (**Figure 3**; **Supplementary Table 7**) revealed: p.R246Q, p.G203S, and p.R246W were significantly associated with a higher likelihood of hypospadias (all *P* < 0.05); p.R227Q was significantly associated with a lower likelihood of cryptorchidism (*P* = 0.001) and scrotal abnormalities (*P* = 0.002); no significant associations were observed between any of the eight mutations and minor phenotypes (all *P*>0.05).

**Figure 3:**
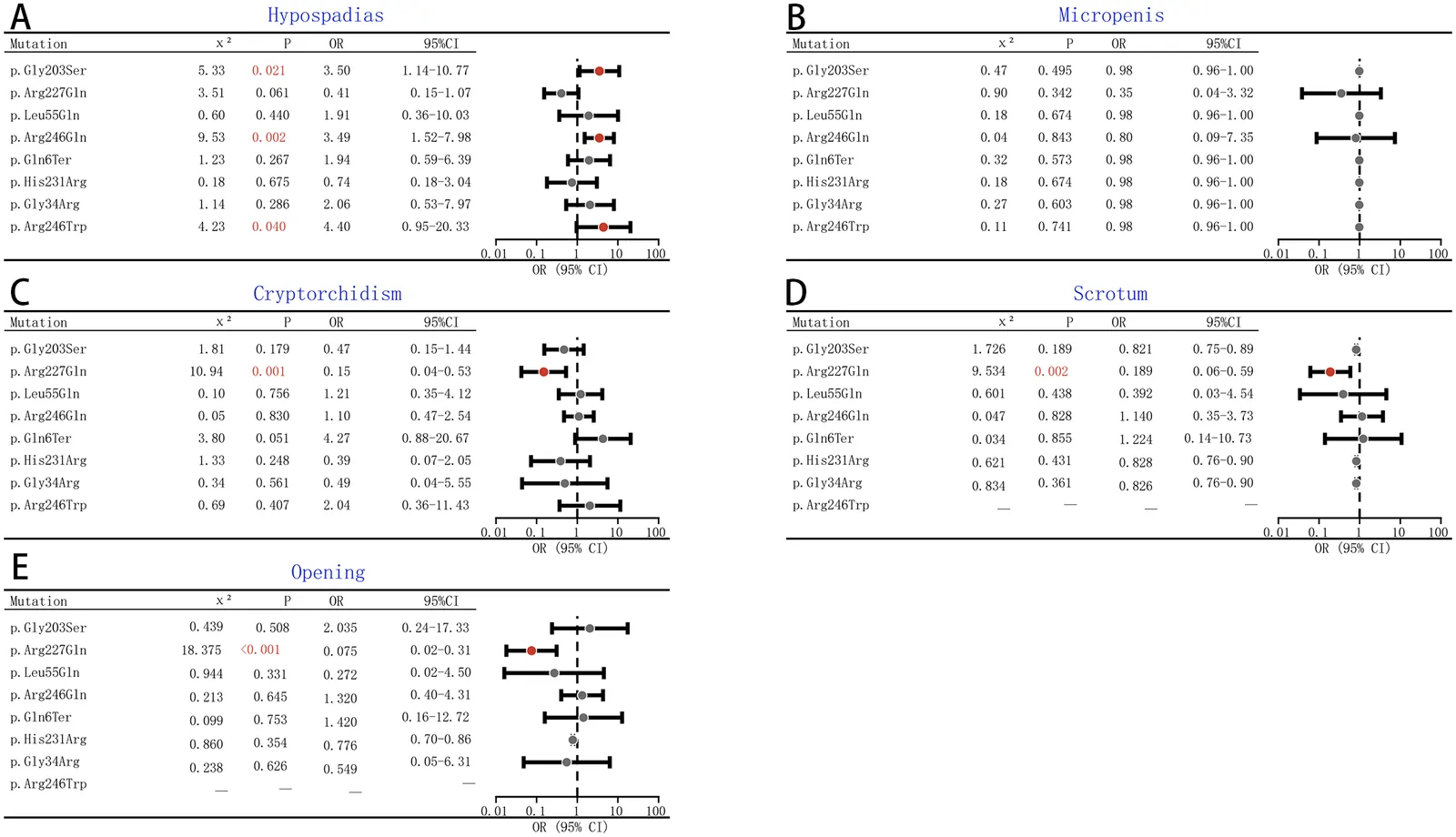
Associations of specific *SRD5A2* mutations with external genital phenotypes. Forest plots show mutation-specific associations with hypospadias **(A)**, micropenis **(B)**, cryptorchidism **(C)**, scrotal abnormalities **(D)**, and urethral/perineal opening **(E)**. Significant results are highlighted in red.

#### 3.4.2 Hormonal profiles by genotype

Kruskal-Wallis H tests stratified by age group (**Table 2**; **Supplementary Table 8**) showed no significant associations between mutation types and serum T, DHT, T/DHT ratio, LH, or E2 (all *P*>0.05). Only in the adolescence and adulthood group, FSH levels (n=35) differed significantly by mutation type (χ²=10.884, *P* = 0.028), with frameshift mutations showing lower FSH than nonsense mutations (corrected *P* = 0.029).

**Table 2:** Association of *SRD5A2* mutation types with hormonal levels across age groups.

Age group	Infancy		Childhood		Adolescence and adulthood	
	M(IQR)	*P*	M(IQR)	*P*	M(IQR)	*P*
T (nmol/L)	2.45(0.29,7.34)	0.344	1.90(0.09,10.40)	0.486	21.00(5.81,23.98)	0.328
DHT (nmol/L)	0.39(0.12,0.82)	0.296	0.49(0.14,1.05)	0.100	0.62(0.40,1.02)	0.399
T/DHT	7.40(1.25,16.60)	0.221	4.50(1.00,12.65)	0.480	32.70(16.90,49.02)	0.820
FSH (IU/L)	1.50(1.00,2.40)	0.229	3.20(0.59,15.30)	0.434	8.56(6.20,14.50)	**0.028**
LH (IU/L)	1.82(0.20,2.85)	0.128	3.60(0.12,7.30)	0.183	7.60(3.90,10.58)	0.217
E2 (pg/ml)	14.00(14.00,14.00)	—	27.40(27.40,27.40)	—	27.47(20.30,41.62)	0.375

The table includes overall *P*-values from Kruskal-Wallis H tests for each hormonal parameter across three age strata (infancy, childhood, and adolescence/adulthood). Bold indicates statistical significance (*P*<0.05).

Mann-Whitney U tests comparing homozygous vs. compound heterozygous patients (**Figure 4**; **Supplementary Table 9**) showed no significant differences in T, DHT, T/DHT ratio, LH, or FSH (all *P* > 0.05).

**Figure 4:**
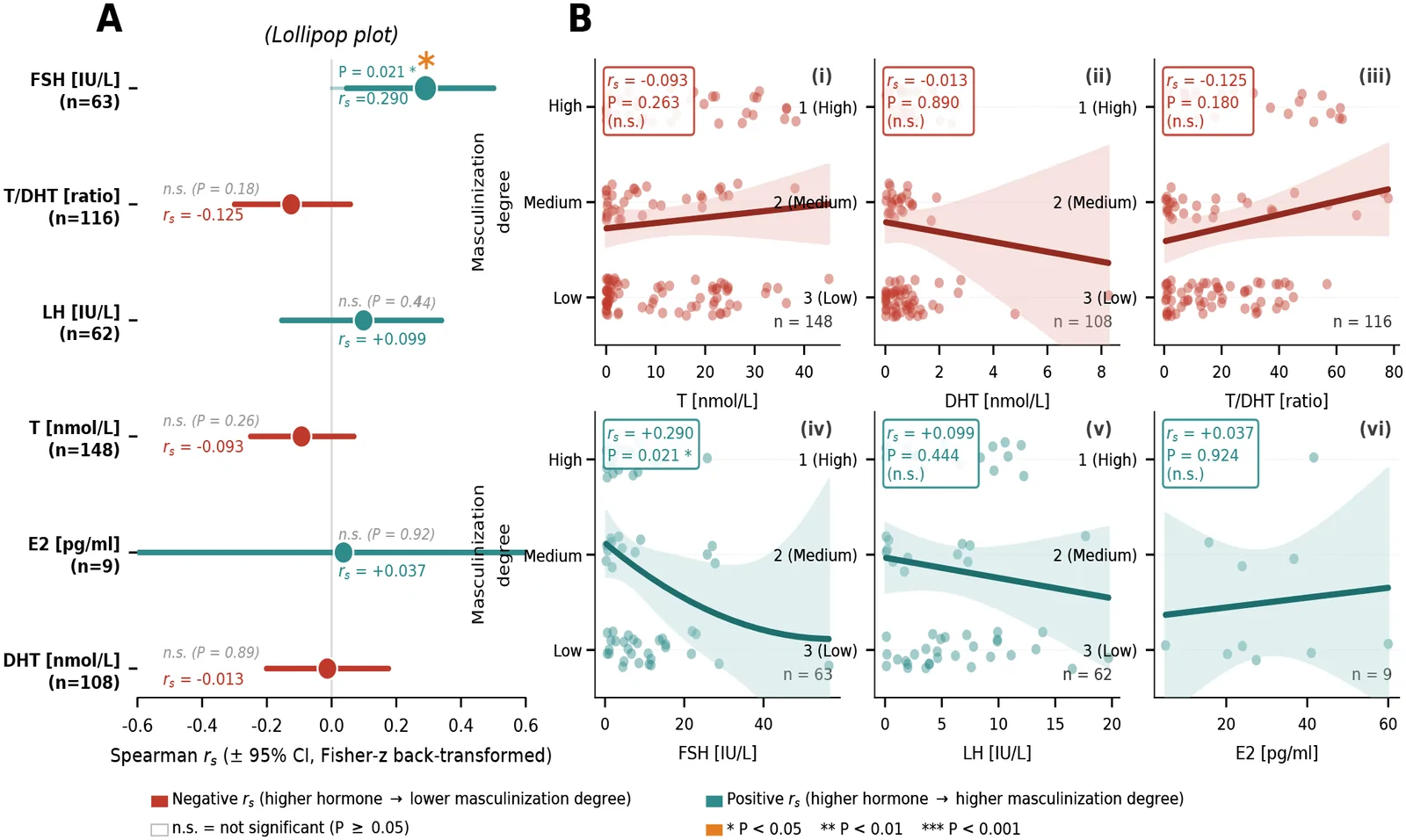
Correlations between hormone levels and external genital masculinization in patients with 5α-reductase type 2 deficiency. **(A)** Lollipop plot showing Spearman rank correlation coefficients (rs) with 95% confidence intervals (Fisher-z back-transformed) between endocrine hormone levels and external genitalia masculinization degree. Red indicates negative and teal indicates positive correlations. FSH is the only hormone significantly correlated with masculinization degree (rs = +0.290, *P* = 0.021). n.s., not significant (*P* ≥ 0.05). **(B)** Scatter plots showing the relationship between individual hormone levels and masculinization degree High, Medium, Low for (i) T, (ii) DHT, (iii) T/DHT ratio, (iv) FSH, (v) LH, and (vi) E2. Fitted curves with 95% confidence bands are shown. Sample sizes (n) are indicated in each panel. *P* < 0.05.

Spearman correlation analysis (**Figure 4**; **Supplementary Table 9**) revealed a significant positive correlation between serum FSH levels and the degree of external genital masculinization (r_s_=0.290, *P* = 0.021).

#### 3.4.3 Growth and pubertal development by genotype

Height SDS and weight SDS did not differ significantly across mutation types (ANOVA: *P* = 0.133 and *P* = 0.130, respectively) (**Supplementary Table 10**).

Tanner stage comparisons (**Supplementary Table 11**) showed significant differences across mutation types (H = 10.778, df=3, *P* = 0.013). Pairwise comparisons revealed that patients with frameshift mutations had significantly lower Tanner stages than compound heterozygotes (corrected *P* = 0.013).

#### 3.4.4 Gender assignment and prognosis by genotype

Initial sex assignment did not differ significantly across mutation types (likelihood ratio χ²=0.374, df=4, *P* = 0.985) (**Supplementary Table 12**). Gender dysphoria incidence (18 patients) showed no significant association with mutation type (likelihood ratio χ²=3.107, df=2, *P* = 0.211).

### 3.5 Phenotype, geography, and diagnosis age

#### 3.5.1 Phenotype and geographic location

Sinnecker grade distribution differed significantly by geographic region (χ²=35.944, df=12, *P* < 0.001; Cramér’s V = 0.268) and by continent (χ²=25.346, df=6, *P* < 0.001; Cramér’s V = 0.231) (**Figure 5**; **Supplementary Table 13**). Feminine phenotypes were more frequent in West Asia/Middle East and North Africa, while masculinized phenotypes predominated in East Asia. Country-level differences were not significant (*P* = 0.211).

**Figure 5:**
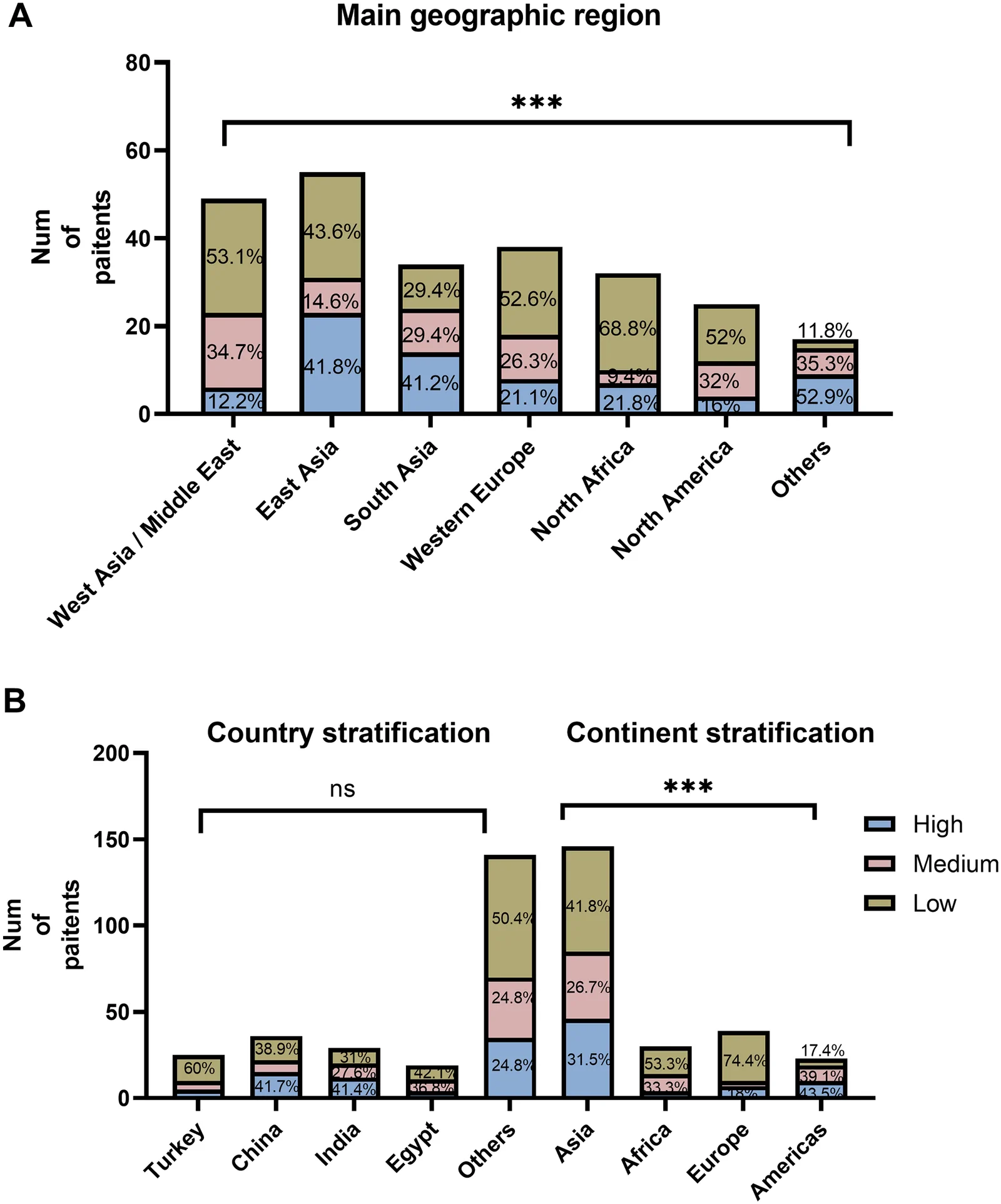
Geographic distribution of external genital masculinization severity in patients with 5α-reductase type 2 deficiency. Stacked bar charts show the proportions of high, medium, and low masculinization across main geographic regions **(A)** and by country and continent stratification **(B)**. Phenotypic severity differed significantly by geographic region and continent, but not by country. ***P < 0.001; ns, not significant.

#### 3.5.2 Phenotype, genotype, and age at diagnosis

Age at diagnosis (months) (**Table 3**; **Supplementary Table 14**) was significantly earlier in compound heterozygous compared to homozygous patients (mean rank: 95.08 vs 116.15, Z=-2.177, *P* = 0.029). However, no significant differences were observed by mutation type (*P* = 0.712) or phenotypic severity (*P* = 0.922). After age stratification (infancy/childhood/adulthood), these associations were not significant, suggesting that zygosity influences diagnostic timing but not categorical age distribution.

**Table 3:** Analysis of the association between genetic mutation characteristics and age at diagnosis.

Analysis type	Mutation group	Statistical methods	Statistic	*df*	*P*
Continuous age (months)	Homozygous/Compound Heterozygous	Mann-Whitney U test	*Z*=-2.177	1	**0.029**
	Different Mutation Type	Kruskal-Wallis H Test	—	—	0.712
Categorized age (stratified)	Homozygous/Compound Heterozygous	Pearson Chi-square test	χ²=1.609	2	0.447
		Test for linear trend	χ²=0.806	1	0.369
		Spearman rank correlation	r_s_=-0.060	—	0.374
	Different Mutation Type	Pearson Chi-square test	χ²=6.214	8	0.623
		Likelihood Ratio Tes	χ²=6.367	8	0.606

Z=Standardized test statistic of Mann-Whitney U test; χ²=Chi-square test statistic; df=Degrees of freedom; *P*=*P* value; r_s_=Spearman rank correlation coefficient; “—”=not applicable. non-parametric tests were used for continuous variables and chi-square tests for categorical variables as age at diagnosis was not normally distributed. Bold *P* values indicate statistically significant differences (*P*<0.05).

### 3.6 Association of consanguinity with genotype and phenotype

Among 172 patients with documented parental marital status, we analyzed consanguinity in relation to zygosity, mutation type, and Sinnecker grade (**Figure 6**; **Supplementary Table 15**).

**Figure 6:**
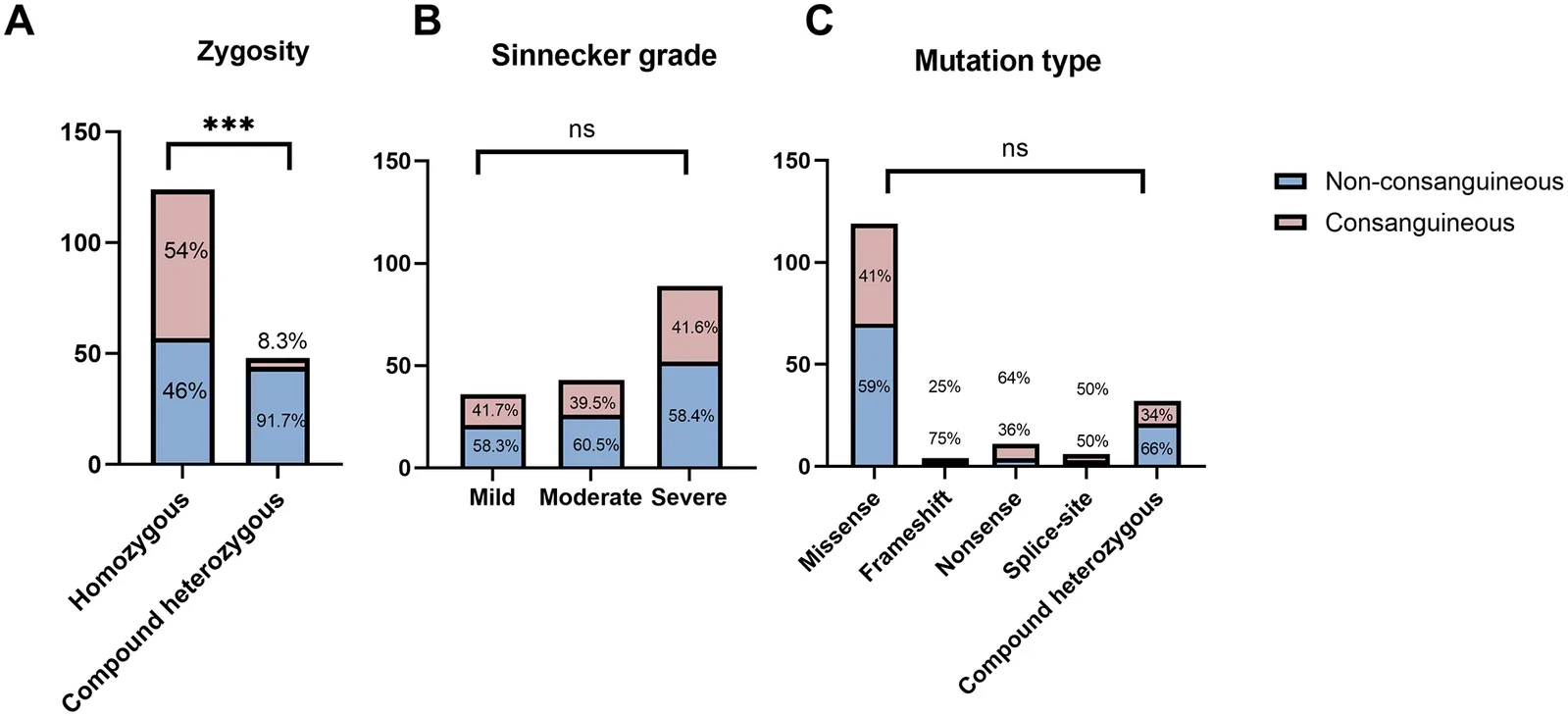
Association of consanguinity with zygosity, mutation type, and phenotypic severity. Consanguinity was significantly associated with homozygous mutations **(A)**, but showed no significant association with Sinnecker phenotypic grade **(B)** or mutation type distribution **(C)**. ***P < 0.001; ns, not significant.

#### 3.6.1 Consanguinity and zygosity

Pearson χ² test revealed a highly significant association between consanguinity and homozygous mutations (χ²=29.814, df=1, *P* < 0.001). In consanguineous families, 94.4% (67/71) were homozygous vs. 56.4% (57/101) in non-consanguineous families.

#### 3.6.2 Consanguinity and phenotypic severity

Ordinal logistic regression showed no significant association between consanguinity and Sinnecker grade (Wald χ²=1.759, *P* = 0.185), with the proportional odds assumption satisfied (parallel line test, *P* = 0.122).

#### 3.6.3 Consanguinity and MUTATION TYPE

No significant association was found between consanguinity and mutation type distribution (χ²=3.524, df=4, *P* = 0.474); multinomial logistic regression confirmed this finding (overall model *P* = 0.475), with no mutation subtype showing significant enrichment in the consanguineous group (all *P*>0.05).

## 4 Discussion

This study, based on a large cohort of 279 genetically confirmed patients with 5α-RD2 from 36 countries, systematically illustrates the core associations between *SRD5A2* mutations and clinical phenotypes, overcoming the limitations of previous small-series studies characterized by contradictory findings and fragmented evidence. Unlike traditional studies that merely catalog genotype–clinical feature correspondences (54, 55), our findings demonstrate that the genotype–phenotype relationship in 5α-RD2 is not a simple linear correlation, but rather a complex regulatory network jointly modulated by mutation molecular characteristics, residual enzymatic activity, endocrine regulation, and geographical population heterogeneity. This mechanistic framework explains the fundamental causes of the marked phenotypic heterogeneity of the disease and provides a novel scientific basis for resolving long-standing controversies in the field and guiding precision clinical management.

### 4.1 Mutation zygosity as the core genetic determinant of phenotypic severity

Our study first established that mutation zygosity status is a core genetic determinant of phenotypic severity. Patients with homozygous mutations exhibited significantly more severe undermasculinization of external genitalia compared to compound heterozygotes (*P* = 0.011) (56). The underlying mechanism of this difference is closely linked to gene dosage effects and compensatory residual enzymatic activity. Homozygous mutations result in complete or near-complete loss of *SRD5A2* function (57), leading to profound depletion of 5α-reductase type 2 activity and near-total blockade of the T-to-DHT conversion pathway (5). During the critical fetal period of external genital masculinization, the absence of effective androgenic stimulation ultimately results in more severe genital developmental abnormalities (58–60). In contrast, compound heterozygous mutations typically involve combinations of two different mild pathogenic variants at distinct loci, which may retain partial residual enzymatic activity (61). Simultaneously, the organism may partially compensate for DHT synthesis defects through compensatory regulatory mechanisms and alternative metabolic pathways (62), thereby attenuating phenotypic damage (14). This finding clarifies the previously puzzling observation that “patients with the same mutation can exhibit variable phenotypes (54)” and confirms that zygosity status is a key genetic marker for stratifying phenotypic severity, providing a core basis for rapid clinical prognostication.

### 4.2 Functional differentiation of mutation subtypes and phenotype-specific

The functional differences among distinct mutation subtypes represent a key molecular mechanism shaping the specific differentiation of clinical phenotypes. Our study confirmed that *SRD5A2* mutations exhibit clear functional stratification and phenotype-specific targeting: p.R246Q, p.G203S, and p.R246W were significantly associated with a higher likelihood of hypospadias; p.R227Q was significantly associated with a lower likelihood of cryptorchidism and scrotal abnormalities; and frameshift mutations were specifically associated with impaired pubertal development.

The essence of these differential phenotypic associations lies in the distinct protein functional domains affected by each mutation site. p.R246Q and p.G203S are localized to the substrate-binding region of the enzyme, directly disrupting testosterone binding and catalytic function, preferentially affecting external genital structural development (14). p.R227Q, located in the NADPH-binding region, mildly reduces catalytic efficiency without completely disrupting protein spatial structure, thereby retaining partial physiological function (14). This mutation may also maintain the stability of the hypothalamic-pituitary-gonadal axis and provides basal DHT, reducing the risk of gonadal developmental anomalies (63). Frameshift mutations, by contrast, lead to protein translation truncation and complete disruption of spatial structure, with near-total loss of enzymatic activity that cannot be rescued by compensatory pathways, ultimately specifically interfering with pubertal developmental processes (64, 65). This explanation is consistent with the previous notion that “high heterogeneity of disease-causing mutant functions (54)”, demonstrating that different *SRD5A2* mutations target distinct developmental pathways, thereby generating characteristic clinical phenotype spectra.

### 4.3 Endocrine modifier effects: FSH as a key mediator of phenotypic severity

The endocrine modifier effects identified in this study further refine the non-linear regulatory system of genotype–phenotype relationships in 5α-RD2. Previous studies have largely focused on gene mutations themselves while neglecting the secondary modulation of phenotype by endocrine parameters. Our study demonstrated that serum FSH levels are significantly positively correlated with phenotypic severity (r_s_=0.290, *P* = 0.021), establishing FSH as a key intermediate regulatory factor connecting genotype to clinical phenotype.

Well known: severe pathogenic mutations impair gonadal feedback, triggering compensatory FSH elevation. FSH normally supports Sertoli cell proliferation, differentiation, and paracrine functions critical for germ cell development (66, 67). In 5α-RD2, however, profound DHT deficiency deprives Sertoli cells of essential androgen signaling. Although FSH rises compensatorily, this elevation cannot restore Sertoli cell function without adequate androgen support; chronic overstimulation may even induce receptor desensitization and dysregulation of Sertoli-derived factors, ultimately impairing spermatogonial development. Thus, higher FSH levels not only reflect the severity of androgen deficiency but also actively contribute to phenotypic severity by exacerbating Sertoli cell dysfunction. This establishes FSH as a key endocrine modifier linking genotype to clinical outcome. Animal studies have demonstrated that the administration of 5α-reductase inhibitors impairs spermatogenesis and alters pituitary feedback mechanisms (68).

Notably, classical diagnostic parameters such as T, DHT, and the T/DHT ratio showed no significant association with phenotypic severity (63, 69, 70), suggesting that traditional endocrine markers are only useful for disease diagnosis and cannot accurately stratify disease severity. The absence of mutation-type-specific T/DHT differences across all age groups suggests that hormonal markers reflect the global enzymatic deficiency but are insufficient to predict individual mutation-specific clinical trajectories. FSH may serve as a novel endocrine indicator for auxiliary assessment of phenotypic severity and prognostication of developmental outcomes, filling a gap in clinical severity stratification. Meanwhile, the lack of significant effects of different mutation types and zygosity status on basal sex hormone levels explains why some patients with similar hormonal profiles can exhibit markedly different clinical phenotypes, further corroborating the multifactorial nature of genotype–phenotype associations.

### 4.4 Geographical population heterogeneity as a macro-level modulator

Geographical population heterogeneity represents a major macro-level factor mediating genotype–phenotype differences and a core reason why prior single-center studies have failed to reach unified conclusions. Our study confirmed that the *SRD5A2* mutation spectrum exhibits significant geographic clustering: p.R246Q, p.G203S, and p.R227Q are predominant mutations in Asian populations; European and American populations harbor characteristic mutations such as p.H231R and p.R246W; and p.G34R is an Africa-specific enriched mutation. Furthermore, the distribution of phenotypes differs markedly across regions, with a higher proportion of feminized phenotypes in West Asia/Middle East and North Africa, and more masculinized phenotypes in East Asia.

These differences are not determined by a single genetic factor but result from the interplay of genetic background (71–73), consanguinity rates (74), regional medical resources (7), and racial genetic polymorphisms (75). Regions with high consanguinity rates exhibit higher proportions of homozygous mutations and more severe phenotypes (74, 76). Meanwhile, racial genetic polymorphisms can modulate residual enzymatic activity and compensatory pathway efficiency, modifying the pathogenic effects of mutations (75, 77, 78). This finding suggests that maybe no universal standard exists for genotype–phenotype associations in 5α-RD2; clinical interpretation of mutation pathogenicity and prognostication must be individualized based on regional population characteristics.

### 4.5 Clinical translational value

The genotype–phenotype associations established in this study carry substantial clinical translational value. At the diagnostic level, the multidimensional association system based on mutation subtype, zygosity status, geographical features, and FSH levels can move beyond the traditional single-model approach of “genetic confirmation equals diagnosis,” achieving an upgrade from disease diagnosis to severity stratification and prognostic prediction.

At the clinical intervention level, patients carrying high-risk mutation subtypes (p.R246Q, p.G203S) require focused screening for hypospadias and advance planning of surgical correction; p.R227Q carriers should prioritize gonadal development assessment to reduce the risk of cryptorchidism-related complications; and patients with frameshift mutations require long-term monitoring of pubertal development and early intervention for developmental delay.

Regarding gender assignment decisions, our study confirmed that mutation type is not directly associated with initial sex assignment or the incidence of gender dysphoria, suggesting that clinical gender decisions should not rely solely on genotype results but should integrate genital phenotype, psychological assessment, and sociocultural factors (79)—correcting the previous misconception of making decisions based on genotype or phenotype alone.

In terms of prognostic assessment, homozygous mutations, elevated FSH levels, and frameshift mutations can serve as high-risk warning indicators of adverse developmental outcomes, providing precise targets for long-term follow-up management.

Furthermore, consanguinity is strongly associated with homozygous mutations (*P* < 0.001), making it a key indicator for prioritized genetic screening in affected families. Although consanguinity does not independently predict phenotype severity, its enrichment of homozygous mutations—which correlate with more severe undermasculinization and delayed diagnosis—underscores the need for early proactive screening and genetic counseling regarding recurrence risks. Therefore, consanguinity status should be incorporated as a routine clinical parameter in diagnostic and management algorithms for 5α-RD2.

### 4.6 Study limitations

Several limitations of this study should be acknowledged. First, EMS and Sinnecker scores were rarely reported directly in the original studies; the vast majority were retrospectively calculated from narrative clinical descriptions. Although we employed dual independent reviewers, standardized scoring templates, and consensus-based adjudication, this approach inevitably introduces some subjectivity and potential classification bias.

Second, the included studies employed heterogeneous hormone assay methods, units, and hCG stimulation protocols. We addressed this through unit conversion, preferential use of post-stimulation T/DHT ratios, age stratification, and non-parametric statistics. The T/DHT ratio partially corrects for inter-assay bias, but the lack of standardization remains challenging. After careful consideration, post-hCG stimulation data were not included in core statistical analyses and are presented descriptively only.

Third, sociocultural factors influencing gender assignment could only be described observationally rather than systematically analyzed, as individual-level sociocultural data were unavailable from the original studies.

Fourth, long-term follow-up data (pubertal development, gender identity, fertility outcomes) were available for only a minority of patients, limiting statistical inference for these endpoints. Among them, the number of cases with documented social gender change (n = 44) and gender dysphoria (n = 18) was relatively small, limiting statistical power.

Fifth, height and weight data were limited (19 and 13 patients, respectively), and these growth parameter findings should be interpreted as exploratory and hypothesis-generating.

Sixth, consanguinity, age at diagnosis, and access to specialized care could not be directly controlled in a multivariate model at the individual level due to the nature of pooled analysis. However, stratified analyses—including comparisons of zygosity, mutation type, and geographic region—were performed to evaluate their potential confounding effects.

Finally, since all patients were genetically confirmed (the gold standard), our core genotype–phenotype associations are unlikely to be substantially confounded. Nevertheless, residual confounding cannot be entirely excluded. Future prospective, multi-center registry studies with standardized data collection are warranted to validate these findings.

### 4.7 Conclusion

In summary, through systematic analysis of the largest cohort of 279 genetically confirmed patients with 5α-RD2, this study demonstrates that the genotype–phenotype relationship in 5α-RD2 is non-linear and multifactorial, jointly modulated by mutation subtype, residual enzymatic activity, zygosity status, hypothalamic-pituitary-gonadal axis feedback status, geographical distribution, consanguinity and medical resource availability. p.R227Q is associated with a lower likelihood of gonadal developmental anomalies, potentially attributable to partial preservation of NADPH-binding function; p.R246Q leads to severe phenotypes through structural destabilization; homozygous mutations result in more severe phenotypes but later diagnosis due to complete enzymatic activity loss; and FSH levels, as an upstream indicator reflecting the severity of androgen deficiency, correlate with phenotypic severity. Consanguinity is strongly associated with homozygous mutations, highlighting its critical role in genetic counseling and early screening strategies. These findings provide more robust evidence for clinical diagnosis, treatment decisions, and genetic counseling in 5α-RD2, and offer important empirical support from large-scale data to substantially advance the understanding over genotype–phenotype associations in this field. Some important results of the above studies are summarized in a graphical abstract, see **Figure 7** for details.

**Figure 7:**
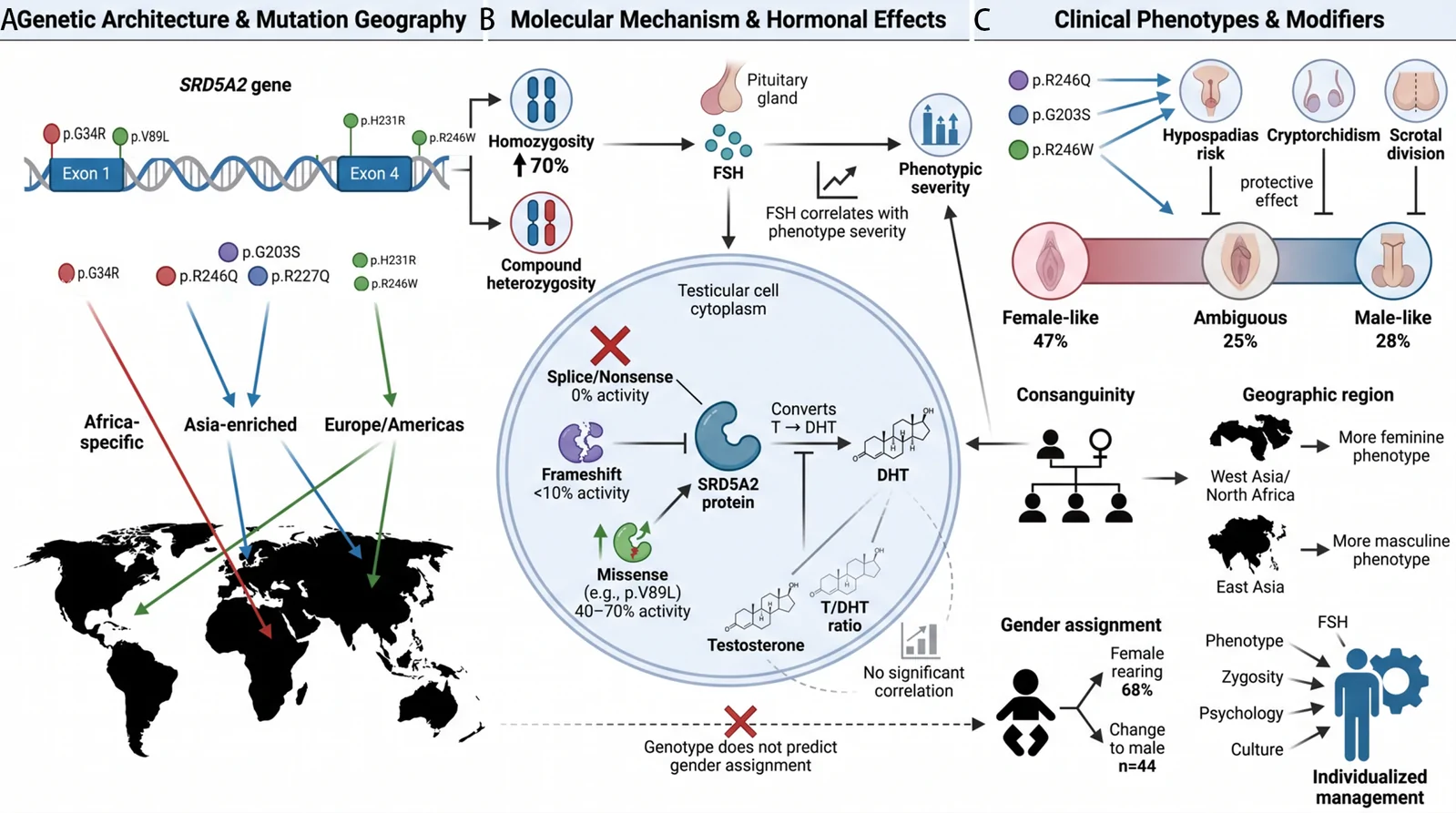
Genotype–phenotype correlations in 5α-reductase type 2 deficiency. Graphical abstract illustrating the genetic architecture, molecular mechanisms, geographic heterogeneity, and clinical implications of 5α-reductase type 2 deficiency. **(A)** *SRD5A2* mutations show distinct genetic architecture and geographic heterogeneity, with recurrent variants enriched in specific populations, including p.G34R in Africa, p.R246Q/p.G203S/p.R227Q in Asia, and p.H231R in Europe/Americas. **(B)** Pathogenic *SRD5A2* variants reduce 5α-reductase type 2 activity and impair testosterone-to-dihydrotestosterone conversion, contributing to variable external genital masculinization; homozygous mutations are associated with more severe undermasculinization, and FSH correlates with phenotypic severity. **(C)** Clinical phenotypes are modified by mutation subtype, zygosity, consanguinity, geographic background, endocrine status, and psychosocial factors. p.R246Q, p.G203S, and p.R246W were significantly associated with a higher likelihood of hypospadias; p.R227Q was significantly associated with a lower likelihood of cryptorchidism and scrotal abnormalities. These findings support individualized diagnosis, counseling, and management rather than genotype-only decision-making.

## Data Availability

The original contributions presented in the study are included in the article/**Supplementary Material**. Further inquiries can be directed to the corresponding authors.
