# Supplementary material for: Clinical characteristics and genotype–phenotype correlation analysis in 279 patients with 5α-reductase type 2 deficiency
Source: Front Endocrinol (Lausanne). 2026 Sep 25;17:1919643. doi: 10.3389/fendo.2026.1919643 (PMC13649436; doi:10.3389/fendo.2026.1919643)
Supplement: Supplementary file 1 [file DataSheet1.pdf]

Supplementary Material

1 EMS and Sinnecker Scale

EMS (External Masculinization Score)[1]

I. Original Definition and Source

The External Masculinization Score (EMS) was developed by Ahmed et al. in 2000 and published in BJU International. This scoring system was designed to provide a standardized, objective quantitative tool for external genitalia assessment. The total score ranges from 0 to 12, with higher scores indicating a greater degree of masculinization.

II. Scoring Components and Criteria

The EMS comprises four dimensions, each scored independently:

Dimension	Scoring Criteria	Score
**Scrotal fusion**	Complete fusion (normal male appearance)	3
	Partial fusion	2
	No fusion (completely separate, female appearance)	0
**Stretched penile length**	≥2.5 cm (normal)	3
	<2.5 cm (micropenis)	2
	Clitoris-like structure only	1
	No penile structure	0
**Position of urethral meatus**	Glanular (normal)	3
	Penile shaft hypospadias	2
	Penoscrotal/perineal hypospadias	1
	Perineal opening (urogenital sinus)	0
**Site of gonads**	Bilateral testes in scrotum	3
	Unilateral in scrotum/inguinal	1.5
	Bilateral non-palpable/intra-abdominal	0

III. Rules for Reconstructing EMS from Narrative Clinical Descriptions

When the original literature did not directly report EMS values, scores were retrospectively calculated according to the following rules based on extracted clinical descriptions:

1. Scrotal fusion (0–3 points)

Description of "complete scrotal fusion," "normal scrotum," "male scrotal appearance" → 3 points

Description of "partial scrotal fusion," "incomplete scrotal fusion," "bifid scrotum," "scrotal cleft" → 2 points

Description of "no scrotal fusion," "completely separated labioscrotal folds," "female external genital appearance," "labia-like structures" → 0 points

2. Stretched penile length (0–3 points)

Explicitly reported stretched length ≥2.5 cm → 3 points

Explicitly reported stretched length <2.5 cm, or described as "micropenis" → 2 points

Described as "clitoris-like phallus," "clitoromegaly," "short but recognizable phallus" → 1 point

Described as "no phallic structure," "only clitoris visible," "absent phallus" → 0 points

3. Position of urethral meatus (0–3 points)

Described as "glanular meatus," "normal urethral position" → 3 points

Described as "penile shaft hypospadias," "meatus on penile shaft" → 2 points

Described as "penoscrotal hypospadias," "perineal hypospadias," "meatus in perineum" → 1 point

Described as "urogenital sinus," "single perineal opening," "no separate urethral meatus identifiable" → 0 points

4. Site of gonads (0–3 points)

Described as "bilateral testes in scrotum," "bilateral scrotal testes palpable" → 3 points

Described as "unilateral testis in scrotum, contralateral non-palpable/inguinal," "bilateral inguinal testes palpable" → 1.5 points

Described as "bilateral non-palpable testes," "bilateral intra-abdominal testes," "testes not visualized" → 0 points

Important notes:

If descriptive information for a given dimension was incomplete or ambiguous, that dimension was recorded as "not assessable" and excluded from total score calculation.

When described as "completely female external genitalia," all four dimensions were assigned the minimum scores (scrotal fusion 0, phallus 0, meatus 0, gonads 0), resulting in EMS = 0.

Sinnecker Scale[2]

I. Original Definition and Source

The Sinnecker scale was developed by Sinnecker et al. in 1996 and published in the American Journal of Medical Genetics. This grading system was specifically designed for phenotypic classification of 5α-reductase type 2 deficiency, categorizing patients into 5 grades (1–5) based on the severity of external genital masculinization defects. Grades 1–4 are further subdivided into a and b subtypes, yielding a total of 9 levels in the full scale.

II. Grading Criteria and Definitions

Grade	Subtype	Description	Clinical Features
Grade 1	1a	Completely male, no evidence of undervirilization	Normal male external genitalia
	1b	Completely male, but impaired spermatogenesis	Normal external genital appearance, may be associated with infertility
Grade 2	2a	Predominantly male, isolated hypospadias	Isolated hypospadias, no other significant abnormalities
	2b	Predominantly male, micropenis with severe hypospadias	Micropenis + severe hypospadias
Grade 3	3a	Ambiguous external genitalia	External genitalia between male and female, indeterminate phenotype
	3b	Ambiguous external genitalia, more female-oriented	More severe limited masculinization, phallic/clitoral structure indeterminate
Grade 4	4a	Predominantly female	Mainly female external appearance with some masculinization features
	4b	Predominantly female, more severe	Severe female-like external genitalia, urogenital sinus with perineal opening
Grade 5	—	Completely female	Completely female external genital appearance

III. Rules for Reconstructing Sinnecker Grade from Narrative Clinical Descriptions

Core principle: Assign the grade based on the degree of external genital masculinization, ranging from the most masculinized (1a) to the most feminized (5).

Step 1 — Determine "completely male" phenotype (Grade 1)

External genitalia entirely normal male appearance → 1a

Normal male external genital appearance but with documented spermatogenic dysfunction/infertility → 1b

Step 2 — Determine "predominantly male" phenotype (Grade 2)

Isolated hypospadias, normal penile size, no other abnormalities → 2a

Micropenis + hypospadias (penoscrotal or more severe) → 2b

If cryptorchidism is present but external genitalia are essentially male, assign to Grade 2

Step 3 — Determine "ambiguous" phenotype (Grade 3)

External genitalia cannot be clearly classified as male or female; some masculinization evident but clearly insufficient → 3a

More severe ambiguity; phallic structure difficult to distinguish between penis and clitoris; associated with urogenital sinus and labioscrotal folds → 3b

Step 4 — Determine "predominantly female" phenotype (Grade 4)

External genitalia mainly female appearance but with some masculinization features (e.g., mild clitoromegaly, partial labial fusion) → 4a

Severe female-like external genitalia; significant clitoromegaly; urogenital sinus with perineal opening → 4b

Step 5 — Determine "completely female" phenotype (Grade 5)

External genitalia entirely female appearance, no evidence of any masculinization → 5

Important notes:

When descriptive information was insufficient to precisely assign an a/b subtype, the case was recorded at the grade level only (e.g., "Grade 3" without specifying 3a/3b).

If contradictory information was present (e.g., "male appearance but partially fused labia"), the most severe masculinization defect was used as the basis for grading.

Cases with insufficient descriptive information for reliable grading were recorded as "not assessable."

2 Supplementary Tables

Sup Table 1 Individual Participant Data (IPD) of All Patients Included in This Study

Study No.	Author, Year	Country	N	Pt ID	SRD5A2 Mutation	Zygosity	Mutation Type
1	Perry, 2011[3]	Pakistan	4	1	c.755+1G>T<br>p.Arg246Gln	Compound heterozygote	Splice-site mutation<br>Missense mutation
				2	c.755+1G>T	Homozygote	Splice-site mutation
				3	c.755+1G>T<br>p.Arg246Gln	Compound heterozygote	Splice-site mutation<br>Missense mutation
				4	c.755+1G>T<br>p.Arg246Gln	Compound heterozygote	Splice-site mutation<br>Missense mutation
2	Parlak, 2014[4]	Turkey	1	5	p.Tyr235Phe	Homozygote	Missense mutation
3	Wigley, 1994[5]	Caucasus	1	6	p.Tyr235Phe	Homozygote	Missense mutation
4	Mazen, 2003[6]	Israel, Egypt	9	7	p.Tyr235Phe	Homozygote	Missense mutation
				8	p.Gly34Arg	Homozygote	Missense mutation
				9	p.Asn160Asp	Homozygote	Missense mutation
				10	p.Gly34Arg	Homozygote	Missense mutation
				11	p.Tyr235Phe	Homozygote	Missense mutation
				12	p.Gly34Arg	Homozygote	Missense mutation
				13	p.Gly34Arg	Homozygote	Missense mutation
				14	p.Asn160Asp	Homozygote	Missense mutation
				15	p.Gly34Arg	Homozygote	Missense mutation
5	Nicoletti, 2005[7]	Italy	2	16	p.Tyr235Phe	Homozygote	Missense mutation
				17	p.Tyr235Phe	Homozygote	Missense mutation
6	Baldinotti, 2008[8]	Italy	1	18	p.Tyr235Phe	Homozygote	Missense mutation
7	Fernández-Cancio, 2004[9]	Spain	1	19	p.Gly115Asp<br>p.Arg246Trp	Compound heterozygote	Missense mutation<br>Missense mutation

8	Balagamage, 2025[10]	India	14	20	p.Leu86Argfs*50<br>p.Cys88Trp	Compound heterozygote	Frameshift mutation<br>Missense mutation
				21	p.Glu200Lys	Homozygote	Missense mutation
				22	p.Gly196Ser<br>p.Arg246Gln	Compound heterozygote	Missense mutation<br>Missense mutation
				23	p.Arg246Gln	Homozygote	Missense mutation
				24	p.Ala228Thr	Homozygote	Missense mutation
				25	p.Glu200Lys	Homozygote	Missense mutation
				26	p.Arg246Gln	Homozygote	Missense mutation
				27	p.Arg246Gln	Homozygote	Missense mutation
				28	p.Glu200Lys	Homozygote	Missense mutation
				29	p.Arg227Ter	Homozygote	Nonsense mutation
				30	p.Glu200Lys<br>p.Gly203Ser	Compound heterozygote	Missense mutation<br>Missense mutation
				31	p.Arg227Ter	Homozygote	Nonsense mutation
				32	p.Arg227Ter	Homozygote	Nonsense mutation
				33	p.Gly196Ser<br>p.Arg246Gln	Compound heterozygote	Missense mutation<br>Missense mutation
9	Chávez, 2000[11]	Mexico	2	34	p.Glu197Asp<br>p.Pro212Arg	Compound heterozygote	Missense mutation<br>Missense mutation
				35	p.Glu197Asp	Homozygote	Missense mutation
10	Viga, 2014[12]	France	1	36	p.Gly115Asp	Homozygote	Missense mutation
11	Sasaki, 2019[13]	Japan	4	37	p.Tyr26Ter<br>p.Arg227Gln	Compound heterozygote	Nonsense mutation<br>Missense mutation
				38	p.Gly34Arg<br>p.Arg227Gln	Compound heterozygote	Missense mutation<br>Missense mutation
				39	p.Arg227Gln	Homozygote	Missense mutation
				40	p.Arg227Gln	Homozygote	Missense mutation

12	Deeb, 2016[14]	UAE	11	41	exon 2 deletion	Homozygote	Deletion mutation
				42	NR	NR	NR
				43	NR	NR	NR
				44	NR	NR	NR
				45	NR	NR	NR
				46	NR	NR	NR
				47	NR	NR	NR
				48	NR	NR	NR
				49	NR	NR	NR
				50	NR	NR	NR
				51	NR	NR	NR
13	Simsek, 2012[15]	Turkey	8	52	p.Arg103Ter	Homozygote	Nonsense mutation
				53	p.Leu55Gln	Homozygote	Missense mutation
				54	p.Tyr91His	Homozygote	Missense mutation
				55	p.Met157del	Homozygote	Deletion mutation
				56	p.Gly203Ser	Homozygote	Missense mutation
				57	p.Gly203Ser	Homozygote	Missense mutation
				58	p.Leu55Gln<br>p.Arg171Ser	Compound heterozygote	Missense mutation<br>Missense mutation
				59	p.Ala65Pro	Homozygote	Missense mutation
14	Ocal, 2002[16]	Turkey	6	60	p.Leu55Gln	Homozygote	Missense mutation
				61	p.Leu55Gln	Homozygote	Missense mutation
				62	p.Leu55Gln	Homozygote	Missense mutation
				63	p.Leu55Gln<br>p.Arg171Ser	Compound heterozygote	Missense mutation<br>Missense mutation
				64	NR	Homozygote	NR

				65	p.Met157del	Homozygote	Deletion mutation
15	Boudon, 1995[17]	Turkey	1	66	p.Met157del	Homozygote	Deletion mutation
16	Bahceci, 2005[18]	Turkey	2	67	p.Gly123Arg	Homozygote	Missense mutation
				68	p.Gly123Arg	Homozygote	Missense mutation
17	Hiort, 1996[19]	NR, Germany, Turkey	9	69	p.Leu55Gln<br>p.Gly196Ser	Compound heterozygote	Missense mutation<br>Missense mutation
				70	p.Leu55Gln	Homozygote	Missense mutation
				71	p.Met157del	Homozygote	Deletion mutation
				72	p.Gly196Ser	Homozygote	Missense mutation
				73	p.Gly196Ser	Homozygote	Missense mutation
				74	p.Arg227Gln	Homozygote	Missense mutation
				75	p.Ala228Thr	Homozygote	Missense mutation
				76	p.His231Arg	Homozygote	Missense mutation
				77	p.Ile112Asn<br>p.Gln126Arg	Compound heterozygote	Missense mutation<br>Missense mutation
18	Can, 1998[20]	Turkey	1	78	A single adenine (A) deletion causes a frameshift at amino acid 251	Homozygote	Frameshift mutation
19	Maimoun, 2010[21]	France, Turkey	4	79	p.Lys41Thrfs*93	Homozygote	Frameshift mutation
				80	p.Gly115Asp	Homozygote	Missense mutation
				81	p.Ala215Val<br>p.Ter255Gln	Compound heterozygote	Missense mutation<br>Missense mutation
				82	p.Ser14Arg<br>p.Leu89Val	Compound heterozygote	Missense mutation<br>Missense mutation
20	Skordis, 2011[22]	Cyprus	1	83	c.281-2A>G	Homozygote	Splice-site mutation
21	Di Marco, 2013[23]	Iraq	7	84	p.Phe151Phefs*8	Homozygote	Frameshift mutation
				85	p.Phe151Phefs*8	Homozygote	Frameshift mutation
				86	p.Phe151Phefs*8	Homozygote	Frameshift mutation
				87	p.Phe151Phefs*8	Homozygote	Frameshift mutation

				88	p.Phe151Phefs*8	Homozygote	Frameshift mutation
				89	p.Tyr91His	Homozygote	Missense mutation
				90	p.Tyr91His	Homozygote	Missense mutation
22	Anwar, 1997[24]	Pakistan	1	91	p.Glu200Lys	Homozygote	Missense mutation
23	Li, 2021[25]	China	1	92	p.Arg227Gln<br>p.Met1Ile	Compound heterozygote	Missense mutation<br>Missense mutation
24	Jia, 2018[26]	China, India, Mexico, South Korea, Thailand	19	93	p.Gln6Ter<br>p.Arg246Gln	Compound heterozygote	Nonsense mutation<br>Missense mutation
				94	p.Gln6Ter<br>p.Arg246Gln	Compound heterozygote	Nonsense mutation<br>Missense mutation
				95	p.Gln6Ter<br>p.Ala228Val	Compound heterozygote	Nonsense mutation<br>Missense mutation
				96	p.Gln6Ter	Homozygote	Nonsense mutation
				97	p.Gln6Ter<br>p.Asn193Ser	Compound heterozygote	Nonsense mutation<br>Missense mutation
				98	p.Gln6Ter<br>p.Gly203Ser	Compound heterozygote	Nonsense mutation<br>Missense mutation
				99	p.Gln6Ter<br>p.Gly203Ser	Compound heterozygote	Nonsense mutation<br>Missense mutation
				100	p.Arg246Gln<br>p.Gly203Ser	Compound heterozygote	Missense mutation<br>Missense mutation
				101	p.Arg246Gln<br>p.Gly203Ser	Compound heterozygote	Missense mutation<br>Missense mutation
				102	p.Arg246Gln<br>p.Cys222PhefsTer232	Compound heterozygote	Missense mutation<br>Frameshift mutation
				103	p.Arg246Gln	Homozygote	Missense mutation
				104	p.Arg246Gln	Homozygote	Missense mutation
				105	p.Arg246Gln	Homozygote	Missense mutation
				106	p.Arg246Gln	Homozygote	Missense mutation
				107	p.Arg246Gln	Homozygote	Missense mutation
				108	p.Arg246Gln	Homozygote	Missense mutation

				109	p.Arg246Gln<br>p.Gly203Ser	Compound heterozygote	Missense mutation<br>Missense mutation
				110	p.Arg246Gln<br>p.Asn193Ser	Compound heterozygote	Missense mutation<br>Missense mutation
				111	p.Arg246Gln<br>p.Gly203Ser	Compound heterozygote	Missense mutation<br>Missense mutation
25	Shabir, 2012[27]	India	1	112	p.Ala52Thr<br>c.281+15T>C	Compound heterozygote	Missense mutation<br>Splice-site mutation
26	Cocchetti, 2020[28]	Caucasian, Italy	2	113	p.Ala207Asp<br>p.Tyr235Phe	Compound heterozygote	Missense mutation<br>Missense mutation
				114	p.Gly183Ser<br>p.Leu111Hisfs*24	Compound heterozygote	Missense mutation<br>Frameshift mutation
27	Ko, 2010[29]	South Korea	6	115	p.Phe219Serfs*58	Homozygote	Frameshift mutation
				116	p.Phe219Serfs*58	Homozygote	Frameshift mutation
				117	p.Arg246Gln	Homozygote	Missense mutation
				118	p.Arg246Gln	Homozygote	Missense mutation
				119	p.Gln6Ter<br>p.Arg246Gln	Compound heterozygote	Nonsense mutation<br>Missense mutation
				120	p.Gly203Ser<br>p.Arg246Gln	Compound heterozygote	Missense mutation<br>Missense mutation
28	Sahakitrungruang, 2008[30]	Thailand	2	121	p.Gln6Ter<br>p.Gly203Ser	Compound heterozygote	Nonsense mutation<br>Missense mutation
				122	p.Leu20Pro<br>p.Gly183Ser	Compound heterozygote	Missense mutation<br>Missense mutation
29	Sahu, 2009[31]	India	3	123	p.Arg246Gln	Homozygote	Missense mutation
				124	p.Arg246Gln	Homozygote	Missense mutation
				125	p.Gln56His<br>p.Leu89Val<br>c.281+15T>C	Compound heterozygote	Missense mutation<br>Missense mutation<br>Splice-site mutation
30	Shabir, 2013[32]	India	2	126	p.Val63Alafs*?	Homozygote	Frameshift mutation
				127	p.Val63Alafs*?	Homozygote	Frameshift mutation
31	Savas, 2010[33]	Turkey	1	128	p.Ala65Pro	Homozygote	Missense mutation
32	Sasaki, 2003[34]	Japan	1	129	p.Gln6Ter	Homozygote	Nonsense mutation

33	Tsai, 2012[35]	China	1	130	p.Gln71Ter<br>p.Arg227Gln	Compound heterozygote	Nonsense mutation<br>Missense mutation
34	Reddy, 2021[36]	India	3	131	p.Arg246Gln	Homozygote	Missense mutation
				132	p.Arg246Gln	Homozygote	Missense mutation
				133	p.Arg246Gln	Homozygote	Missense mutation
35	Dalili, 2020[37]	Iran	1	134	p.Ile159Arg	Homozygote	Missense mutation
36	Chen, 2023[38]	NR	1	135	NR	NR	NR
37	Akiba, 2021[39]	China	1	136	p.Ala217Glu	Homozygote	Missense mutation
38	Yang, 2020[40]	China	1	137	p.Arg227Gln	Homozygote	Missense mutation
39	Maimoun, 2011[41]	African, France, Tunisia, Turkey	4	138	p.Leu55Gln	Homozygote	Missense mutation
				139	p.Gln56Arg	Homozygote	Missense mutation
				140	p.Asn193Ser	Homozygote	Missense mutation
				141	p.Ala12Glnfs*?	Compound heterozygote	Frameshift mutation<br>Missense mutation
40	Li, 2018[42]	China	1	142	p.His232Arg<br>p.Gln6Ter	Compound heterozygote	Missense mutation<br>Nonsense mutation
41	Chan, 2009[43]	China	5	143	p.Leu55Pro<br>p.Arg227Gln	Compound heterozygote	Missense mutation<br>Missense mutation
				144	p.Gly203Ser	Homozygote	Missense mutation
				145	p.Gln6Ter<br>p.Arg227Gln	Compound heterozygote	Nonsense mutation<br>Missense mutation
				146	p.Arg227Gln	Homozygote	Missense mutation
				147	p.Arg227Gln<br>p.Val10Gly	Compound heterozygote	Missense mutation
42	Walter, 2010[44]	Iraq	1	148	p.Leu55Gln	Homozygote	Missense mutation
43	Rafigh, 2022[45]	Iran	1	149	p.Tyr242Asp	Homozygote	Missense mutation
44	Hiort, 2002[46]	Caucasian	1	150	p.Arg111Ter	Homozygote	Nonsense mutation
45	Le, 2022[47]	Vietnam	1	151	p.His162Pro<br>p.Arg227Gln	Compound heterozygote	Missense mutation<br>Missense mutation
46	Fénichel, 2013[48]	NR	4	152	p.Gly34Arg	Homozygote	Missense mutation
				153	p.Asn193Ser	Homozygote	Missense mutation

				154	Exon 1 complete deletion	Homozygote	Gene deletion
				155	p.Arg227Ter<br>p.Ala228Thr	Compound heterozygote	Nonsense mutation<br>Missense mutation
47	Fernández-Cancio, 2004[49]	China	1	156	p.Arg227Gln	Compound heterozygote	Frameshift mutation<br>Missense mutation
48	Kim, 2006[50]	South Korea	2	157	p.Phe219Serfs*58	Homozygote	Frameshift mutation
				158	p.Phe219Serfs*58	Homozygote	Frameshift mutation
49	Ji, 2017[51]	China	1	159	p.Arg227Gln	Homozygote	Missense mutation
50	Avendaño, 2020[52]	NR, China, Venezuela	9	160	p.Asn193Ser	Homozygote	Missense mutation
				161	p.Asn193Ser	Homozygote	Missense mutation
				162	p.Asn193Ser	Homozygote	Missense mutation
				163	p.Gln126Arg<br>p.Asn193Ser	Compound heterozygote	Missense mutation<br>Missense mutation
				164	p.Asn193Ser	Homozygote	Missense mutation
				165	p.Gln6Ter<br>p.Asn193Ser	Compound heterozygote	Nonsense mutation<br>Missense mutation
				166	p.Asn193Ser	Homozygote	Missense mutation
				167	p.Asn193Ser	Homozygote	Missense mutation
				168	p.Arg246Gln<br>p.Asn193Ser	Compound heterozygote	Missense mutation<br>Missense mutation
51	Kumar, 2025[53]	India	3	169	p.Arg246Gln	Homozygote	Missense mutation
				170	p.Arg246Gln	Homozygote	Missense mutation
				171	p.Arg246Gln	Homozygote	Missense mutation
52	Ferraz, 1999[54]	Brazil	1	172	p.Gln126Arg<br>p.Trp140Glyfs*?	Compound heterozygote	Missense mutation<br>Frameshift mutation
53	Rawal, 2025[55]	India	2	173	p.Arg246Gln	Homozygote	Missense mutation
				174	p.Arg246Gln	Homozygote	Missense mutation
54	Eunice, 2008[56]	India	2	175	p.Arg246Gln	Homozygote	Missense mutation
				176	p.Arg246Gln	Homozygote	Missense mutation

55	Wall, 2024[57]	Thailand	1	177	p.Tyr128Ter<br>p.Gly203Ser	Compound heterozygote	Nonsense mutation<br>Missense mutation
56	Bertelloni, 2019[58]	Italy	2	178	p.Arg103Pro<br>p.His230Pro	Compound heterozygote	Missense mutation<br>Missense mutation
				179	p.Arg103Pro<br>p.His230Pro	Compound heterozygote	Missense mutation<br>Missense mutation
57	Hochberg, 1996[59]	Lebanon	5	180	p.Leu55Gln	Homozygote	Missense mutation
				181	p.Leu55Gln	Homozygote	Missense mutation
				182	p.Leu55Gln	Homozygote	Missense mutation
				183	p.Leu55Gln	Homozygote	Missense mutation
				184	p.Leu55Gln	Homozygote	Missense mutation
58	Laureano, 2024[60]	Brazil	2	185	p.Gly183Ser	Homozygote	Missense mutation
				186	p.Gly183Ser	Homozygote	Missense mutation
59	Matsubara, 2010[61]	Japan, Dominican Republic, Sweden	12	187	p.Arg246Gln	Homozygote	Missense mutation
				188	p.Arg246Trp	Homozygote	Missense mutation
				189	p.Arg246Trp	Homozygote	Missense mutation
				190	p.Arg246Trp	Homozygote	Missense mutation
				191	p.Arg246Trp	Homozygote	Missense mutation
				192	p.Arg246Trp	Homozygote	Missense mutation
				193	p.Arg246Trp	Homozygote	Missense mutation
				194	p.Arg246Trp	Homozygote	Missense mutation
				195	p.Arg246Trp	Homozygote	Missense mutation
				196	p.Arg246Trp	Homozygote	Missense mutation
				197	p.His231Arg<br>p.Gly196Ser	Compound heterozygote	Missense mutation<br>Missense mutation
				198	p.His231Arg<br>p.Gly196Ser	Compound heterozygote	Missense mutation<br>Missense mutation

60	Sinnecker, 1996[62]	Eritrea, Germany, Turkey, Vietnam	9	199	p.Ile112Asn<br>p.Gln126Arg	Compound heterozygote	Missense mutation<br>Missense mutation
				200	p.His231Arg	Homozygote	Missense mutation
				201	p.Met157del	Homozygote	Deletion mutation
				202	p.Met157del	Homozygote	Deletion mutation
				203	p.Ala228Thr	Homozygote	Missense mutation
				204	p.Gly196Ser	Homozygote	Missense mutation
				205	p.Gly196Ser	Homozygote	Missense mutation
				206	p.Arg227Gln	Homozygote	Missense mutation
				207	p.Arg227Gln	Homozygote	Missense mutation
61	Vilchis, 2000[63]	Italy, Mexico	5	208	p.Arg246Gln	Homozygote	Missense mutation
				209	p.Gly115Asp	Homozygote	Missense mutation
				210	p.Pro212Arg	Homozygote	Missense mutation
				211	p.Gly85Asp<br>p.Gly115Asp	Compound heterozygote	Missense mutation<br>Missense mutation
				212	p.Ser245Tyr	Homozygote	Missense mutation
62	Mehta, 2021[64]	India	1	213	p.Glu57Ter<br>p.Gly196Ser	Compound heterozygote	Nonsense mutation<br>Missense mutation
63	Zhu, 2014[65]	China	11	214	p.Leu89Val<br>p.Phe219Serfs*58	Compound heterozygote	Missense mutation<br>Frameshift mutation
				215	p.Gly203Ser<br>p.Arg227Gln	Compound heterozygote	Missense mutation<br>Missense mutation
				216	c.IVS4+2T>C	Homozygote	Splice-site mutation
				217	p.Arg246Gln	Homozygote	Missense mutation
				218	p.Arg246Gln<br>p.Asn193Ser	Compound heterozygote	Missense mutation<br>Missense mutation
				219	p.Gly203Ser<br>p.Arg246Gln	Compound heterozygote	Missense mutation<br>Missense mutation

				220	p.Gly203Ser<br>c.281-2A>G	Compound heterozygote	Missense mutation<br>Splice-site mutation
				221	p.Gly203Ser<br>c.281-2A>G	Compound heterozygote	Missense mutation<br>Splice-site mutation
				222	p.Ala228Val	Homozygote	Missense mutation
				223	p.Ala228Val	Homozygote	Missense mutation
				224	p.Gln6Ter<br>p.Gly203Ser	Compound heterozygote	Nonsense mutation<br>Missense mutation
64	Maimoun, 2011[66]	Algeria, Belgium, Cyprus, Egypt, France, India, Italy, Mongolia, Morocco, Poland, Saudi Arabia, Spain, Tunisia, Turkey, Vietnam, Cape Verde, Palestine, Côte d'Ivoire	52	225	p.Gly34Arg	Homozygote	Missense mutation
				226	p.Gly34Arg	Homozygote	Missense mutation
				227	p.Gly34Arg	Homozygote	Missense mutation
				228	p.Gly34Arg	Homozygote	Missense mutation
				229	p.Gly34Arg	Homozygote	Missense mutation
				230	p.Lys41Thrfs*93	Homozygote	Frameshift mutation
				231	p.Leu55Gln	Homozygote	Missense mutation
				232	p.Leu55Gln	Homozygote	Missense mutation
				233	p.Gln56Arg	Homozygote	Missense mutation
				234	p.Gln56Arg	Homozygote	Missense mutation
				235	p.Glu57Gln	Homozygote	Missense mutation
				236	p.Ala62Glu	Homozygote	Missense mutation
				237	p.Ala62Glu	Homozygote	Missense mutation
				238	p.Ala62Glu	Homozygote	Missense mutation
				239	p.Tyr91His	Homozygote	Missense mutation

240	p.Tyr91His	Homozygote	Missense mutation
241	c.281-2A>G	Homozygote	Splice-site mutation
242	c.281-2A>G	Homozygote	Splice-site mutation
243	c.281-2A>G	Homozygote	Splice-site mutation
244	p.Gly115Asp	Homozygote	Missense mutation
245	p.Gly115Asp	Homozygote	Missense mutation
246	p.Gly115Asp	Homozygote	Missense mutation
247	p.Gln126Arg	Homozygote	Missense mutation
248	p.Gln126Arg	Homozygote	Missense mutation
249	p.Gln126Arg	Homozygote	Missense mutation
250	p.Met157del	Homozygote	Deletion mutation
251	p.Met157del	Homozygote	Deletion mutation
252	p.Asn160Asp	Homozygote	Missense mutation
253	p.Asn160Asp	Homozygote	Missense mutation
254	p.Asn193Ser	Homozygote	Missense mutation
255	p.Gly203Ser	Homozygote	Missense mutation
256	p.Gly203Ser	Homozygote	Missense mutation
257	p.Arg227Gln	Homozygote	Missense mutation
258	p.His231Arg	Homozygote	Missense mutation
259	p.Tyr235Phe	Homozygote	Missense mutation
260	p.Arg246Trp	Homozygote	Missense mutation
261	p.Arg246Trp	Homozygote	Missense mutation
262	p.Arg246Trp	Homozygote	Missense mutation

				263	p.Ala12Glnfs*?<br>p.Arg246Trp	Compound heterozygote	Frameshift mutation<br>Missense mutation
				264	p.Gln6Ter<br>p.Arg227Gln	Compound heterozygote	Nonsense mutation<br>Missense mutation
				265	p.Leu55Gln<br>p.Arg171Ser	Compound heterozygote	Missense mutation<br>Missense mutation
				266	p.Gln56Arg<br>c.548-2A>C	Compound heterozygote	Missense mutation<br>Splice-site mutation
				267	p.Gly104Glu<br>p.Ile154Ilefs*?	Compound heterozygote	Missense mutation<br>Frameshift mutation
				268	p.Gly115Asp<br>p.Ala207Asp	Compound heterozygote	Missense mutation<br>Missense mutation
				269	c.281-2A>G<br>p.Arg171Ser	Compound heterozygote	Splice-site mutation<br>Missense mutation
				270	c.281-2A>G<br>p.Pro181Leu	Compound heterozygote	Splice-site mutation<br>Missense mutation
				271	p.Asn193Ser	Uncertain	Missense mutation/?
				272	p.Gly196Ser<br>p.Phe223Ser	Compound heterozygote	Missense mutation<br>Missense mutation
				273	p.Gly203Ser<br>p.His231Arg	Compound heterozygote	Missense mutation<br>Missense mutation
				274	p.Gly203Ser<br>p.His231Arg	Compound heterozygote	Missense mutation<br>Missense mutation
				275	p.Gly203Ser<br>p.His231Arg	Compound heterozygote	Missense mutation<br>Missense mutation
				276	p.Ala215Val<br>p.Ter255Gln	Compound heterozygote	Missense mutation<br>Missense mutation
65	Yao, 2020[67]	China	1	277	p.Arg227Ter<br>p.Gln6Ter	Compound heterozygote	Nonsense mutation<br>Missense mutation
66	Vilchis, 1997[68]	Mexico	2	278	p.Arg227Ter	Homozygote	Nonsense mutation
				279	p.Arg227Ter	Homozygote	Nonsense mutation
	**TOTAL**		**279**				

Note: Study No. = sequential study identifier; Author, Year = first author and year of publication, corresponding to the reference list in the main manuscript; Country = country of patient origin; N = total number of patients reported in each study; Pt ID = patient identifier within each study; SRD5A2 Mutation = specific nucleotide and/or amino acid changes identified in the SRD5A2 gene; Zygosity = homozygous or compound heterozygous; Mutation Type = functional classification including missense, nonsense, frameshift, splice-site, or deletion mutations.Patients within each study are listed consecutively. Blank cells in the Study No., Author, Country, and N columns indicate that the patient belongs to the most recent study listed above. NR = not reported. Total patient count: 279. Total study entries: 66.

Sup Table 2 Geographic distribution of included patients with 5α-RD2 by continent, subregion, and country

Part A. Distribution by continent and subregion

Continents	Subregion	*n*	Frequency(%)
**Asia**	**West Asia / Middle East**	68	25.76
	**East Asia**	55	20.83
	**South Asia**	40	15.15
	**Southeast Asia**	8	3.03
**Europe**	**Western Europe**	34	12.88
	**Southern Europe**	5	1.89
	**Eastern Europe**	2	0.76
**Africa**	**North Africa**	25	9.47
	**West Africa**	2	0.76
	**East Africa**	1	0.38
**Americas**	**North America**	18	6.82
	**South America**	6	2.27
**Total**		264	100.00

Part B. Distribution by country

No.	Country	*n*	Frequency(%)	No.	Country	*n*	Frequency(%)
**1**	**Turkey**	39	14.77	**19**	**Tunisia**	3	1.14
**2**	**China**	36	13.64	**20**	**Venezuela**	3	1.14
**3**	**India**	35	13.56	**21**	**Spain**	3	1.14
**4**	**Egypt**	19	7.20	**22**	**Germany**	3	1.14
**5**	**France**	14	5.30	**23**	**Caucasus**	3	1.14
**6**	**South Korea**	12	4.55	**24**	**Sweden**	2	0.76
**7**	**United Arab Emirates**	11	4.17	**25**	**Morocco**	2	0.76
**8**	**Mexico**	9	3.41	**26**	**Poland**	2	0.76
**9**	**Dominican Republic**	9	3.41	**27**	**Iran**	2	0.76
**10**	**Italy**	8	3.03	**28**	**Israel**	1	0.38
**11**	**Iraq**	8	3.03	**29**	**Eritrea**	1	0.38
**12**	**Japan**	6	2.27	**30**	**Algeria**	1	0.38
**13**	**Pakistan**	5	1.89	**31**	**Cape Verde**	1	0.38
**14**	**Lebanon**	5	1.89	**32**	**Palestine**	1	0.38
**15**	**Cyprus**	5	1.89	**33**	**Belgium**	1	0.38
**16**	**Thailand**	4	1.51	**34**	**Côte d'Ivoire**	1	0.38
**17**	**Vietnam**	4	1.51	**35**	**Mongolia**	1	0.38
**18**	**Brazil**	3	1.14	**36**	**Saudi Arabia**	1	0.38
**Total**						**264**	**100.00**

Note: n=number of cases. Countries are sorted in descending order of patient count. "Caucasus region" includes Georgia, Armenia, and Azerbaijan, which were combined for analysis as individual country data were not available in the original literature.15 patients (5.38%) lacked geographic information and were excluded. The subregional classification was based on the geographic regions defined by the World Health Organization (WHO).

Sup Table 3 Correlation analysis of height and weight SDS with age

**Analytical Items**	***n***	***t***	***P*(t-test)**	**95%*CI***	***r*/r_s**	***P*(correlation)**
**Height SDS One-Sample t-Test**	19	1.067	0.300	-0.350~1.072	—	—
**Correlation between height and age SDS**	19	—	—	—	-0.488	**0.034**
**Weight SDS One-Sample t-Test**	13	-0.483	0.638	-0.100~0.640	—	—
**Correlation between weight and age SDS**	13	—	—	—	-0.133	0.665

Note: SDS = Standard Deviation Score; CI = Confidence Interval; r = Pearson product-moment correlation coefficient; r_s = Spearman rank correlation coefficient; "—" = not applicable. A one-sample t-test was employed to compare the patient's SDS with the reference mean (0) of age-matched, sex-matched normal controls. Spearman rank correlation analysis was used for height-age correlation assessments, and Pearson's correlation coefficient was applied for weight-age correlation analysis. Complete height data were available for 19 patients, and complete weight data were available for 13 patients.

Sup Table 4 Geographic distribution differences of *SRD5A2* gene mutations

Mutation	Asia vs non-Asia		Africa vs non-Africa		Europe vs non-Europe		America vs non-America	
	OR	*P*	*OR*	*P*	*OR*	*P*	*OR*	*P*
**p.Arg246Gln**	11.038	**<0.001**	0.823	**0.012**	0.815	**0.003**	0.461	0.296
**p.Gly203Ser**	4.118	**0.017**	0.916	0.098	0.965	0.956	0.918	0.144
**p.Arg227Gln**	13.375	**0.001**	0.920	0.107	0.916	0.054	0.922	0.154
**p.Leu55Gln**	1.103	**0.001**	0.936	0.153	0.933	0.087	0.937	0.206
**p.Gln6Ter**	10.288	**0.006**	0.936	0.153	0.933	0.087	0.937	0.206
**p.His231Arg**	0.926	**<0.001**	0.968	0.319	1.242	**<0.001**	0.969	0.379
**p.Gly34Arg**	0.052	**<0.001**	61.750	**<0.001**	0.950	0.142	0.953	0.277
**p.Arg246Trp**	0.095	**<0.001**	0.944	0.183	1.629	0.465	30.000	**<0.001**

Note: P=P value; OR=Odds Ratio; All comparisons were performed using the Chi-square test. Bolded P values indicate statistically significant differences ($P<0.05$). An $OR>1$ suggests an enrichment of the mutation in the target population, while an $OR<1$ indicates an enrichment in the non-target population.

Sup Table 5 Association analysis of gene mutation characteristics with external genitalia masculinization (n=201)

Mutation Group	Statistical Methods	Statistic	*df*	*P*
**Homozygous/Heterozygous**	Mann-Whitney U test	Z=−2.532	—	**0.011**
**Different Mutation Types**	Kruskal-Wallis H Test	χ^2=9.942	4	**0.041**

Note: χ^2=Chi-square test statistic; P=P value; r_s=Spearman's rank correlation coefficient; "—"=not applicable. Bolded P values indicate statistically significant differences (P<0.05). The Mann-Whitney U test was used to compare phenotypic severity between homozygous and compound heterozygous mutations. The Kruskal-Wallis H test was employed to analyze overall differences in phenotypic severity among different mutation types, with Bonferroni correction applied for post-hoc pairwise comparisons.

Sup Table 6 Pairwise comparisons with Bonferroni correction for the severity of external genital phenotype among patients with different mutation types

Comparison Group	Rank Sum	*Z*	Unadjusted *P*	Adjusted *P*
**Missense vs splice**	-59.254	--2.637	**0.008**	0.084
**Splice vs compound heterozygous**	50.394	2.107	**0.035**	0.351
**Missense vs nonsense**	-26.129	-1.334	0.182	1.000
**Missense vs frameshift**	-21.396	-1.416	0.157	1.000
**Missense vs compound heterozygous**	-8.860	-0850	0.396	1.000
**Nonsense vs frameshift**	4.732	0.198	0.843	1.000
**Nonsense vs splice**	-33.125	-1.138	0.255	1.000
**Nonsense vs compound heterozygous**	17.269	0.813	0.416	1.000
**Frameshift vs splice**	-37.857	-1.440	0.150	1.000
**Frameshift vs compound heterozygous**	12.537	0.729	0.466	1.000

Note: Z=Standardized test statistic; P=P value. Bonferroni correction was applied for multiple comparisons, adjusting the significance threshold to α=0.005 (0.05/10). Bolded P values indicate statistically significant differences before correction.

Sup Table 7 Analysis of the association between specific *SRD5A2* gene mutations and core external genital phenotypes

Mutation	Hypospadias		Micropenis		Cryptorchidism		Scrotum		Opening	
	OR	***P***	***OR***	***P***	***OR***	***P***	***OR***	***P***	***OR***	***P***
**p.Gly203Ser**	3.504	**0.021**	0.977	0.495	0.473	0.179	0.821	0.189	2.035	0.508
**p.Arg227Gln**	0.405	0.061	0.353	0.342	0.150	**0.001**	0.189	**0.002**	0.075	**<0.001**
**p.Leu55Gln**	1.905	0.440	0.978	0.674	1.213	0.756	0.392	0.438	0.272	0.331
**p.Arg246Gln**	3.488	**0.002**	0.800	0.843	1.096	0.830	1.140	0.828	1.320	0.645
**p.Gln6Ter**	1.942	0.267	0.978	0.573	4.273	0.051	1.224	0.855	1.420	0.753
**p.His231Arg**	0.740	0.675	0.978	0.674	0.387	0.248	0.828	0.431	0.776	0.354
**p.Gly34Arg**	2.060	0.286	0.978	0.603	0.495	0.561	0.826	0.361	0.549	0.626
**p.Arg246Trp**	4.400	**0.040**	0.979	0.741	2.043	0.407	—	—	—	—

Note: P = P value; OR = Odds Ratio; "—" indicates that no relevant data are available. All comparisons were performed using the Pearson Chi-square test, with P values in bold indicating statistical significance (P<0.05). OR>1 suggests that the mutation is associated with an increased risk of the corresponding phenotype, whereas OR<1 suggests a decreased risk for the corresponding phenotype.

Sup Table 8 Dunn’s multiple comparisons for FSH levels in the adolescence and adulthood group

**Comparison Group**	**Unadjusted *P***	**Bonferroni-adjusted *P***
**Frameshift vs. splice**	0.905	1.000
**Frameshift vs. missense**	0.111	1.000
**Frameshift vs. compound heterozygous**	0.124	1.000
**Frameshift vs. nonsense**	**0.003**	**0.029**
**Splice vs. missense**	0.312	1.000
**Splice vs. compound heterozygote**	0.319	1.000
**Splice vs. nonsense**	**0.026**	0.260
**Missense vs. compound heterozygous**	0.986	1.000
**Missense vs. nonsense**	**0.014**	0.136
**Nonsense vs. compound heterozygous**	**0.020**	0.204

Note: The table includes Dunn′s post-hoc comparisons for FSH levels in the adolescence and adulthood group. All values are P-values; bold indicates statistical significance ($P<0.05$).

Sup Table 9 Endocrine profiles by zygosity and correlation with phenotype

Part A. Comparison of endocrine hormone levels between homozygous and compound heterozygous mutations (n=220)

**Hormone parameter**	**Homozygous (n=161)**	**Compound Heterozygous (n=59)**	***Z***	***P***
**T (nmol/L)**	21.50(18.03-24.96)	2.57(-3.93-9.07)	-1.412	0.158
**DHT (nmol/L)**	1.99(1.06-2.92)	0.34(0.07-0.61)	-0.535	0.593
**T/DHT**	10.50(7.93-13.07)	7.40(-13.08-27.88)	-1.394	0.163
**FSH (IU/L)**	12.40(7.85-16.95)	2.00(0.43-3.57)	-1.706	**0.088**
**LH (IU/L)**	7.30(4.35-10.25)	3.30(-1.05-7.65)	0.315	0.753
**E2 (pg/ml)**	27.44(10.96-43.92)	23.90(10.68-37.13)	-0.878	0.435

Part B. Spearman rank correlation analysis between endocrine hormone levels and external genitalia masculinization

**Hormone Parameter**	r_s	***P***	***n***
**T (nmol/L)**	-0.093	0.263	148
**DHT (nmol/L)**	-0.013	0.890	108
**T/DHT**	-0.125	0.180	116
**FSH (IU/L)**	0.290	**0.021**	63
**LH (IU/L)**	0.099	0.444	62
**E2 (pg/ml)**	0.037	0.924	9

Note: T, Testosterone; DHT, Dihydrotestosterone; FSH, Follicle-Stimulating Hormone; LH, Luteinizing Hormone; E2, Estradiol; Z, Standardized test statistic of Mann-Whitney U test; r_s, Spearman rank correlation coefficient; P, P-value; n, Sample size. All values are presented as median (interquartile range, P25–P75). All endocrine parameters were non-normally distributed (Kolmogorov-Smirnov test, all $P<0.001$). Differences between homozygous and compound heterozygous groups were analyzed using the Mann-Whitney U test. Correlations between hormone levels and external genital masculinization were assessed using Spearman rank correlation analysis. Bold P-value indicates statistically significant difference ($P<0.05$).

Sup Table 10 Height and weight SDS by mutation type

Mutation Type	Height SDS (*n*)	Mean rank	Weight SDS (*n*)	Mean rank
**Missense Mutation**	8	9.88	5	6.70
**Nonsense Mutation**	2	8.50	1	1.00
**Frameshift Mutation**	4	14.50	4	10.50
**Splicing Mutation**	1	17.00	0	—
**Compound Heterozygote**	4	4.75	3	4.83
**Total**	**19**	—	**13**	—
**Kruskal-Wallis H test**		*H*=7.753, *P*=0.101		*H*=6.617, *P*=0.085

Note: SDS=Standard Deviation Score. One-way ANOVA results were consistent (height: P=0.133; weight: P=0.130).

Sup Table 11 Comparison of Tanner stages among different mutation types

Mutation Type	*n*	Mean rank of Tanner staging
**Missense Mutation**	11	13.36
**Nonsense Mutation**	2	8.00
**Frameshift Mutation**	4	3.50
**Compound Heterozygous Mutation**	6	16.50
**Overall test**	***H*=10.778**	***df*=3，*P*=0.013**

Bonferroni correction pairwise comparisons

Comparison group	Unadjusted *P*	Bonferroni-adjusted *P*
**Frameshift vs nonsense**	0.428	1.000
**Frameshift vs missense**	0.010	0.060
**Frameshift vs. compound heterozygous**	**0.002**	**0.013**
**Nonsense vs missense**	0.287	1.000
**Nonsense vs compound heterozygous**	0.112	0.673
**Missense vs compound heterozygous**	0.346	1.000

Note: Tanner stage = Pubertal development stage; n = sample size; H = Kruskal-Wallis H statistic; df = degrees of freedom; P = P value. Statistically significant differences are indicated by bold P values ($P < 0.05$). The Kruskal-Wallis H test was utilized for group comparisons, as Tanner stage development is an ordinal categorical variable. Post hoc pairwise comparisons were conducted using Bonferroni correction. Mean ranks are derived from the Kruskal-Wallis H test results.

Sup Table 12 Initial rearing gender distribution and gender anxiety incidence among different SRD5A2 mutation types

Part A. Initial rearing gender distribution by mutation type (n=229)

Mutation Type	*n*	Initial female rearing gender [*n* (%)]	Initial male rearing gender [*n* (%)]
**Missense Mutation**	156	103(66.0)	53(34.0)
**Nonsense Mutation**	10	7(70.0)	3(30.0)
**Frameshift Mutation**	13	9(69.2)	4(30.8)
**Splicing Mutation**	6	4(66.7)	2(33.3)
**Compound heterozygous**	44	31(70.5)	13(29.5)
**Total**	**229**	**154(67.2)**	**75(32.8)**

Part B. Gender dysphoria by mutation type (n=18)

Mutation Type	*n*	No gender anxiety [*n*(%)]	Gender anxiety [*n*(%)]
**Missense Mutation**	12	5(41.7)	7(58.3)
**Frameshift Mutation**	2	2(100.0)	0(0.0)
**Splicing**	0	—	—
**Compound heterozygous mutation**	4	2(50.0)	2(50.0)
**Total**	**18**	**9(50.0)**	**9(50.0)**

Note: n, Number of cases; (%), Proportion. For Part A, 4 cells (40.0%) had expected counts < 5 (minimum expected count = 1.97), so the likelihood ratio chi-square test was used for group comparison. No statistically significant difference was observed in initial rearing gender distribution among different mutation types (likelihood ratio $\chi^2 = 0.374$, df = 4, P = 0.985). For Part B, 4 cells (66.7%) had expected counts < 5 (minimum expected count = 1.00). Fisher's exact test was not feasible due to software limitations, so the likelihood ratio chi-square test was used as an alternative. No statistically significant difference was found in the incidence of gender anxiety and identity disorder among different mutation types (likelihood ratio $\chi^2 = 3.107$, df = 2, P = 0.211).

Sup Table 13 External genital masculinization degree by geographic region

Geographic classification	Masculinization Degree [*n*(%)]			Overall test results				
	High	Medium	Low	χ^2	*df*	*P*	*V*	*P*
**Main geographic region**								
**West Asia / Middle East**	6(12.2)	17(34.7)	26(53.1)	—	—	—	—	—
**East Asia**	23(41.8)	8(14.5)	24(43.6)	—	—	—	—	—
**South Asia**	14(41.2)	10(29.4)	10(29.4)	—	—	—	—	—
**Western Europe**	8(21.1)	10(26.3)	20(52.6)	—	—	—	—	—
**North Africa**	7(21.9)	3(9.4)	22(68.8)	—	—	—	—	—
**North America**	4(16.0)	8(32.0)	13(52.0)	—	—	—	—	—
**Others**	9(52.9)	6(35.3)	2(11.8)	—	—	—	—	—
**Total**	71(28.4)	62(24.8)	117(46.8)	35.944	12	**<0.001**	0.268	**<0.001**
**Country stratification**								
**Turkey**	5(20.0)	5(20.0)	15(60.0)	—	—	—	—	—
**China**	15(41.7)	7(19.4)	14(38.9)	—	—	—	—	—
**India**	12(41.4)	8(27.6)	9(31.0)	—	—	—	—	—
**Egypt**	4(21.1)	7(36.8)	8(42.1)	—	—	—	—	—
**Others**	35(24.8)	35(24.8)	71(50.4)	—	—	—	—	—
**Total**	71(28.4)	62(24.8)	117(46.8)	10.842	8	0.211	0.147	0.211
**Continent stratification**								
**Asia**	46(31.5)	39(26.7)	61(41.8)	—	—	—	—	—
**Africa**	4(13.3)	10(33.3)	16(53.3)	—	—	—	—	—
**Europe**	7(17.9)	3(7.7)	29(74.4)	—	—	—	—	—
**Americas**	10(43.5)	9(39.1)	4(17.4)	—	—	—	—	—
**Total**	67(28.2)	61(25.6)	110(46.2)	25.346	6	**<0.001**	0.231	**<0.001**

Note: n=Number of cases; (%)=Proportion ratio; χ^2=Pearson chi-square test statistic; df=Degrees of freedom; P=P value; V=Cramer's V coefficient. The degree of masculinization was classified based on the Sinnecker scale: Low (grades 4a–5, predominantly female external genitalia), Medium (grades 3a–3b, ambiguous external genitalia), and High (grades 1a–2b, predominantly male external genitalia). The Pearson chi-square test was used to analyze the distribution differences of masculinization degree across different regions, and Cramer's V coefficient measured the strength of the association. Bolded P values indicate statistically significant differences ($P<0.05$). A Cramer's V value of 0.1-0.3 suggests a weak to moderate association.

Sup Table 14 Association analysis of external genitalia masculinization degree with age at diagnosis (n=202)

Analysis type	Statistical Methods	Statistic	*df*	*P*
**Continuous age (months)**	Kruskal-Wallis H Test	—	2	0.922
	Spearman rank correlation	r_s=-0.027	—	0.706
**Categorized age (stratified)**	Pearson Chi-square test	χ^2=1.702	4	0.790
	Test for linear trend	χ^2=0.377	1	0.539
	Spearman rank correlation	r_s=0.048	—	0.496

Note: χ^2=Chi-square test statistic; df=Degrees of freedom; P=P value; r_s=Spearman rank correlation coefficient; "—"=not applicable. Kruskal-Wallis H test and Spearman rank correlation were used for continuous variables, and chi-square test and linear-by-linear association test were used for categorical variables as age at diagnosis was not normally distributed.

Sup Table 15 Association of consanguinity with zygosity, mutation type, and phenotypic severity

Analysis	Non-consanguineous	Consanguineous
Consanguinity vs. zygosity(n=172)	n=101	n=71
Homozygous	57 (56.4%)	67 (94.4%)
Compound heterozygous	44 (43.6%)	4 (5.6%)
		Test : Pearson χ^2 ***P* : <0.001**
Consanguinity vs. Sinnecker grade(n=168)	n=99	n=69
Mild	21 (21.2%)	15 (21.7%)
Moderate	26 (26.3%)	17 (24.6%)
Severe	52 (52.5%)	37 (53.6%)
		Test : Ordinal logistic regression *P* : 0.185
Consanguinity vs. mutation type(n=172)	n=101	n=71
Missense	70 (69.3%)	49 (69.0%)
Frameshift	3 (3.0%)	1 (1.4%)
Nonsense	4 (4.0%)	7 (9.9%)
Splice-site	3 (3.0%)	3 (4.2%)
Compound heterozygous	21 (20.8%)	11 (15.5%)
		Test : R×C χ^2 *P* : 0.474

Note: Based on 172 patients with documented parental marital status (71 consanguineous, 101 non-consanguineous); Sinnecker grade analysis included 168 patients with complete phenotypic data (69 consanguineous, 99 non-consanguineous). Categorical data are presented as n (%). Bold P indicates statistical significance ($P<0.05$).

3 Reference

[1] S.F. Ahmed, O. Khwaja, and I.J.B.i. Hughes, The role of a clinical score in the assessment of ambiguous genitalia. 85 (2000) 120-124.

[2] G.H. Sinnecker, O. Hiort, E. Nitsche, P.-M. Holterhus, K. Kruse, and G.C.I.S.J.E.j.o.p. Group, Functional assessment and clinical classification of androgen sensitivity in patients with mutations of the androgen receptor gene. 156 (1996) 7-14.

[3] R.J. Perry, E. Novikova, A.M. Wallace, and M.D. Donaldson, Pitfalls in the diagnosis of 5α-reductase type 2 deficiency during early infancy. Hormone research in paediatrics 75 (2011) 380-2.

[4] M. Parlak, E. Durmaz, S. Gursoy, I. Bircan, and S. Akcurin, Try235Phe homozygous mutation of the steroid 5-a reductase type 2 (SRD5A2) gene in a Turkish patient. Annals of Saudi medicine 34 (2014) 254-6.

[5] W.C. Wigley, J.S. Prihoda, I. Mowszowicz, B.B. Mendonca, M.I. New, J.D. Wilson, and D.W. Russell, Natural mutagenesis study of the human steroid 5 alpha-reductase 2 isozyme. Biochemistry 33 (1994) 1265-70.

[6] I. Mazen, Y.Z. Gad, M. Hafez, C. Sultan, and S. Lumbroso, Molecular analysis of 5alpha-reductase type 2 gene in eight unrelated egyptian children with suspected 5alpha-reductase deficiency: prevalence of the G34R mutation. Clinical endocrinology 58 (2003) 627-31.

[7] A. Nicoletti, L. Baldazzi, A. Balsamo, L. Barp, P. Pirazzoli, M. Gennari, G. Radetti, E. Cacciari, and A. Cicognani, SRD5A2 gene analysis in an Italian population of under-masculinized 46,XY subjects. Clinical endocrinology 63 (2005) 375-80.

[8] F. Baldinotti, S. Majore, A. Fogli, G. Marrocco, P. Ghirri, M. Vuerich, S. Tumini, B. Boscherini, M. Vetri, S. Scommegna, R. Rinaldi, P. Simi, and P. Grammatico, Molecular characterization of 6 unrelated Italian patients with 5alpha-reductase type 2 deficiency. Journal of andrology 29 (2008) 20-8.

[9] M. Fernández-Cancio, J. Rodó, P. Andaluz, M.J. Martínez de Osaba, F. Rodríguez-Hierro, C. Esteban, A. Carrascosa, and L. Audí, Clinical, biochemical and morphologic diagnostic markers in an infant male pseudohermaphrodite patient with compound heterozygous mutations (G115D/R246W) in SRD5A2 gene. Hormone research 62 (2004) 259-64.

[10] C. Balagamage, R. Igbokwe, T. Cole, E.S. Baranowski, L. McCarthy, H. Chandran, E. Van Der Merwe, P. Fulton, C. Godber, J. Kirk, Z. Mohamed, and J. Idkowiak, The diagnostic value of stimulated androgen ratios in 5-alpha reductase type 2 (SRD5A2) deficiency: a case series and review of the literature. Journal of pediatric endocrinology & metabolism : JPEM 38 (2025) 922-930.

[11] B. Chávez, E. Valdez, and F. Vilchis, Uniparental disomy in steroid 5alpha-reductase 2 deficiency. The Journal of clinical endocrinology and metabolism 85 (2000) 3147-50.

[12] L. Vija, S. Ferlicot, D. Paun, H. Bry-Gauillard, G. Berdan, I. Abd-Alsamad, M. Lombès, and J. Young, Testicular histological and immunohistochemical aspects in a post-pubertal patient with 5 alpha-reductase type 2 deficiency: case report and review of the literature in a perspective of evaluation of potential fertility of these patients. BMC endocrine disorders 14 (2014) 43.

[13] G. Sasaki, T. Ishii, N. Hori, N. Amano, K. Homma, S. Sato, and T. Hasegawa, Effects of pre- and post-pubertal dihydrotestosterone treatment on penile length in 5α-reductase type 2 deficiency. Endocrine journal 66 (2019) 837-842.

[14] A. Deeb, H. Al Suwaidi, F. Ibukunoluwa, and S. Attia, Phenotype, Sex of Rearing, Gender Re-Assignment, and Response to Medical Treatment in Extended Family Members with a Novel Mutation in the SRD5A2 Gene. Journal of clinical research in pediatric endocrinology 8 (2016) 236-40.

[15] E. Simsek, C. Binay, and S. Ceylaner, The earlier described mutation (c.307c > T [p.R103X]) in the SRD5A2 gene causing a 46,XY female phenotype. Journal of pediatric endocrinology & metabolism : JPEM 25 (2012) 543-5.

[16] G. Ocal, P. Adiyaman, M. Berberoğlu, E. Cetinkaya, N. Akar, A. Uysal, T. Duman, O. Evliyaoğlu, Z. Aycan, S. Lumbroso, and C. Sultan, Mutations of the 5alpha-steroid reductase type 2 gene in six Turkish patients from unrelated families and a large pedigree of an isolated Turkish village. Journal of pediatric endocrinology & metabolism : JPEM 15 (2002) 411-21.

[17] C. Boudon, J.M. Lobaccaro, S. Lumbroso, G. Ogur, G. Ocal, C. Belon, and C. Sultan, A new deletion of the 5 alpha-reductase type 2 gene in a Turkish family with 5 alpha-reductase deficiency. Clinical endocrinology 43 (1995) 183-8.

[18] M. Bahceci, A.R. Ersay, A. Tuzcu, O. Hiort, A. Richter-Unruh, and D. Gokalp, A novel missense mutation of 5-alpha reductase type 2 gene (SRD5A2) leads to severe male pseudohermaphroditism in a Turkish family. Urology 66 (2005) 407-10.

[19] O. Hiort, G.H. Sinnecker, H. Willenbring, A. Lehners, A. Zöllner, and D. Struve, Nonisotopic single strand conformation analysis of the 5 alpha-reductase type 2 gene for the diagnosis of 5 alpha-reductase deficiency. The Journal of clinical endocrinology and metabolism 81 (1996) 3415-8.

[20] S. Can, Y.S. Zhu, L.Q. Cai, Q. Ling, M.D. Katz, S. Akgun, C.H. Shackleton, and J. Imperato-McGinley, The identification of 5 alpha-reductase-2 and 17 beta-hydroxysteroid dehydrogenase-3 gene defects in male pseudohermaphrodites from a Turkish kindred. The Journal of clinical endocrinology and metabolism 83 (1998) 560-9.

[21] L. Maimoun, P. Philibert, B. Cammas, F. Audran, C. Pienkowski, F. Kurtz, C. Heinrich, M. Cartigny, and C. Sultan, Undervirilization in XY newborns may hide a 5α-reductase deficiency: report of three new SRD5A2 gene mutations. International journal of andrology 33 (2010) 841-7.

[22] N. Skordis, C. Shammas, E. Efstathiou, A. Sertedaki, V. Neocleous, and L. Phylactou, Late diagnosis of 5alpha steroid-reductase deficiency due to IVS12A>G mutation of the SRD5a2 gene in an adolescent girl presented with primary amenorrhea. Hormones (Athens, Greece) 10 (2011) 230-5.

[23] C. Di Marco, A.L. Bulotta, C. Varetti, L. Dosa, A. Michelucci, F. Baldinotti, D. Meucci, C. Castagnini, C. Lo Rizzo, G. Di Maggio, P. Simi, F. Mari, S. Bertelloni, A. Renieri, and M. Messina, Ambiguous external genitalia due to defect of 5-α-reductase in seven Iraqi patients: prevalence of a novel mutation. Gene 526 (2013) 490-3.

[24] R. Anwar, S.G. Gilbey, J.P. New, and A.F. Markham, Male pseudohermaphroditism resulting from a novel mutation in the human steroid 5 alpha-reductase type 2 gene (SRD5A2). Molecular pathology : MP 50 (1997) 51-2.

[25] M. Li, F. Che, S. Qiu, and Z. Wang, [Identification of a novel variant of SRD5A2 gene in a child featuring steroid 5α-reductase type 2 deficiency]. Zhonghua yi xue yi chuan xue za zhi = Zhonghua yixue yichuanxue zazhi = Chinese journal of medical genetics 38 (2021) 1233-1236.

[26] W. Jia, D. Zheng, L. Zhang, C. Li, X. Zhang, F. Wang, Q. Guan, L. Fang, J. Zhao, and C. Xu, Clinical and molecular characterization of 5α-reductase type 2 deficiency due to mutations (p.Q6X, p.R246Q) in SRD5A2 gene. Endocrine journal 65 (2018) 645-655.

[27] I. Shabir, E. Marumudi, M.L. Khurana, and R. Khadgawat, Novel mutation of SRD5A2 gene in a patient with 5α-reductase 2 deficiency from India. BMJ case reports 2012 (2012).

[28] C. Cocchetti, J. Ristori, F. Mazzoli, A. Prunas, S. Bertelloni, A. Magini, L. Vignozzi, M. Maggi, and A.D. Fisher, 5α-Reductase-2 deficiency: is gender assignment recommended in infancy? Two case-reports and review of the literature. Journal of endocrinological investigation 43 (2020) 1131-1136.

[29] J.M. Ko, C.K. Cheon, G.H. Kim, S.H. Kim, K.S. Kim, and H.W. Yoo, Clinical characterization and analysis of the SRD5A2 gene in six Korean patients with 5alpha-reductase type 2 deficiency. Hormone research in paediatrics 73 (2010) 41-8.

[30] T. Sahakitrungruang, S. Wacharasindhu, P. Yeetong, T. Snabboon, K. Suphapeetiporn, and V. Shotelersuk, Identification of mutations in the SRD5A2 gene in Thai patients with male pseudohermaphroditism. Fertility and sterility 90 (2008) 2015.e11-5.

[31] R. Sahu, R. Boddula, P. Sharma, V. Bhatia, R. Greaves, S. Rao, M. Desai, A. Wakhlu, S. Phadke, M. Shukla, P. Dabadghao, R.N. Mehrotra, and E. Bhatia, Genetic analysis of the SRD5A2 gene in Indian patients with 5alpha-reductase deficiency. Journal of pediatric endocrinology & metabolism : JPEM 22 (2009) 247-54.

[32] I. Shabir, M.L. Khurana, E. Marumudi, R. Khadgawat, and A.C. Ammini, Novel nucleotide insertions in two unrelated Indian patients with 5α reductase 2 deficiency leading to premature termination of SRD5A2 enzyme. Steroids 78 (2013) 1159-63.

[33] S. Savas Erdeve, Z. Aycan, M. Berberoglu, Z. Siklar, B. Hacihamdioglu, K. Sipahi, N. Akar, and G. Ocal, A novel mutation of 5alpha-steroid reductase 2 deficiency (CD 65 ALA-PRO) with severe virilization defect in a Turkish family and difficulty in gender assignment. European journal of pediatrics 169 (2010) 991-5.

[34] G. Sasaki, K. Nakagawa, A. Hashiguchi, T. Hasegawa, T. Ogata, and M. Murai, Giant seminoma in a patient with 5 alpha-reductase type 2 deficiency. The Journal of urology 169 (2003) 1080-1.

[35] M.C. Tsai, Y.Y. Chou, S.J. Lin, and L.P. Tsai, A novel SRD5A2 mutation in a Taiwanese newborn with ambiguous genitalia. The Kaohsiung journal of medical sciences 28 (2012) 231-5.

[36] D.S. Reddy, V.S.R. Danda, and S.R. Paidipally, Phenotype variation among siblings with 5-alpha reductase deficiency: A case series. Indian journal of urology : IJU : journal of the Urological Society of India 37 (2021) 183-186.

[37] S. Dalili, B. Rabbani, A. Hassanzadeh Rad, S. Koohmanaee, and N. Mahdieh, A novel pathogenic variant of SRD5A2 in an Iranian psuedohermaphrodite male. Clinical case reports 8 (2020) 1947-1951.

[38] J. Chen, Z. Zhang, W. Shi, Q. Yan, X. Bi, P. Zhu, D. Zhang, and X. Wu, Preimplantation genetic testing and prenatal diagnosis in a family with pseudovaginal perineoscrotal hypospadias: A case report. Medicine 102 (2023) e36171.

[39] K. Akiba, K. Aso, Y. Hasegawa, and M. Fukami, Genome analyses and androgen quantification for an infant with 5α-reductase type 2 deficiency. Journal of pediatric endocrinology & metabolism : JPEM 34 (2021) 1191-1195.

[40] Y.Y. Yang, M. Wang, Q.H. Guo, W.J. Gu, Z.H. Lyu, J.T. Dou, and Y.M. Mu, [A case of 5α-reductase-2 deficiency with small penis and cryptorchidism]. Zhonghua nei ke za zhi 59 (2020) 810-813.

[41] L. Maimoun, P. Philibert, P. Bouchard, G. Ocal, B. Leheup, P. Fenichel, N. Servant, F. Paris, and C. Sultan, Primary amenorrhea in four adolescents revealed 5α-reductase deficiency confirmed by molecular analysis. Fertility and sterility 95 (2011) 804.e1-5.

[42] S.P. Li, L.W. Li, M.X. Sun, X.X. Chen, X.F. Wang, Z.K. Li, S.Y. Zhou, D.C. Zhai, S.X. Geng, S.J. Li, and X.W. Dou, Identification of a novel mutation in the SRD5A2 gene of one patient with 46,XY disorder of sex development. Asian journal of andrology 20 (2018) 518-519.

[43] A.O. Chan, B.W. But, G.T. Lau, A.L. Lam, K.L. Ng, Y.Y. Lam, C.Y. Lee, and C.C. Shek, Diagnosis of 5alpha-reductase 2 deficiency: a local experience. Hong Kong medical journal = Xianggang yi xue za zhi 15 (2009) 130-5.

[44] K.N. Walter, F.B. Kienzle, A. Frankenschmidt, O. Hiort, S.A. Wudy, N. van der Werf-Grohmann, A. Superti-Furga, and K.O. Schwab, Difficulties in diagnosis and treatment of 5alpha-reductase type 2 deficiency in a newborn with 46,XY DSD. Hormone research in paediatrics 74 (2010) 67-71.

[45] M. Rafigh, A. Salmaninejad, B. Sorouri Khorashad, A. Arabi, S. Milanizadeh, M. Hiradfar, and M.R. Abbaszadegan, Novel Deleterious Mutation in Steroid-5α-Reductase-2 in 46, XY Disorders of Sex Development: Case Report Study. Fetal and pediatric pathology 41 (2022) 141-148.

[46] O. Hiort, S.M. Schütt, M. Bals-Pratsch, P.M. Holterhus, C. Marschke, and D. Struve, A novel homozygous disruptive mutation in the SRD5A2-gene in a partially virilized patient with 5alpha-reductase deficiency. International journal of andrology 25 (2002) 55-8.

[47] P.T.Q. Le, T.N.U. Le, T.T.B. Nguyen, M.T. Nguyen, and T.M.T. Ha, An Extremely Rare SRD5A2 Gene c.485A>C Mutation in a Compound Heterozygous Newborn with Disorders of Sex Development First Identified in Vietnam. Case reports in endocrinology 2022 (2022) 6025916.

[48] P. Fénichel, F. Paris, P. Philibert, S. Hiéronimus, L. Gaspari, J.Y. Kurzenne, P. Chevallier, S. Bermon, N. Chevalier, and C. Sultan, Molecular diagnosis of 5α-reductase deficiency in 4 elite young female athletes through hormonal screening for hyperandrogenism. The Journal of clinical endocrinology and metabolism 98 (2013) E1055-9.

[49] M. Fernández-Cancio, M. Nistal, R. Gracia, M.A. Molina, J.A. Tovar, C. Esteban, A. Carrascosa, and L. Audí, Compound heterozygous mutations in the SRD5A2 gene exon 4 in a male pseudohermaphrodite patient of Chinese origin. Journal of andrology 25 (2004) 412-6.

[50] S.H. Kim, K.S. Kim, G.H. Kim, B.M. Kang, and H.W. Yoo, A novel frameshift mutation in the 5alpha-reductase type 2 gene in Korean sisters with male pseudohermaphroditism. Fertility and sterility 85 (2006) 750.e9-750.e12.

[51] W. Ji, L.Y. Zhang, F.C. Li, Y. Wang, W. He, Q.Q. Yin, and Z.H. Liao, Genetic sequencing of a patient with Kallmann syndrome plus 5α-reductase type 2 deficiency. Asian journal of andrology 19 (2017) 386-387.

[52] A. Avendaño, M. González-Coira, I. Paradisi, A. Rojas, G. Da Silva, R. Gómez-Pérez, and J.O. Ceballos, 5α-Reductase type 2 deficiency in families from an isolated Andean population in Venezuela. Annals of human genetics 84 (2020) 151-160.

[53] A. Kumar, A. Kumar, S. Rai, A. Arora, A. Wander, and A. Munshi, Molecular Characterization of Steroid 5 Alpha-Reductase 2 (SRD5A2) Gene Variant in Indian Patients with Disorder of Sexual Development. Archives of sexual behavior 54 (2025) 2755-2761.

[54] L.F. Ferraz, M.T. Mathias Baptista, A.T. Maciel-Guerra, G.G. Júnior, and C. Hackel, New frameshift mutation in the 5alpha-reductase type 2 gene in a Brazilian patient with 5alpha-reductase deficiency. American journal of medical genetics 87 (1999) 221-5.

[55] L. Rawal, D. Panwar, R. Kumar, G. Sharma, S. Jangra, R. Nakra, V. Lal, and V.K. Thamtam, Identification of a Rare Variant in the SRD5A2 Gene in Siblings With 46,XY Disorders of Sexual Development. Case reports in genetics 2025 (2025) 5546459.

[56] M. Eunice, P. Philibert, B. Kulshreshtha, F. Audran, F. Paris, M.L. Khurana, P.E. Pulikkanath, K. Kucheria, C. Sultan, and A.C. Ammini, Molecular diagnosis of 5alpha-reductase-2 gene mutation in two Indian families with male pseudohermaphroditism. Asian journal of andrology 10 (2008) 815-8.

[57] E. Wall, and V. Jayadev, 5α Reductase Deficiency-a Rare Cause of Ambiguous Genitalia and Gender Dysphoria. JCEM case reports 2 (2024) luae191.

[58] S. Bertelloni, F. Baldinotti, G.I. Baroncelli, M.A. Caligo, and D. Peroni, Paternity in 5α-Reductase-2 Deficiency: Report of Two Brothers with Spontaneous or Assisted Fertility and Literature Review. Sexual development : genetics, molecular biology, evolution, endocrinology, embryology, and pathology of sex determination and differentiation 13 (2019) 55-59.

[59] Z. Hochberg, R. Chayen, N. Reiss, Z. Falik, A. Makler, M. Munichor, A. Farkas, H. Goldfarb, N. Ohana, and O. Hiort, Clinical, biochemical, and genetic findings in a large pedigree of male and female patients with 5 alpha-reductase 2 deficiency. The Journal of clinical endocrinology and metabolism 81 (1996) 2821-7.

[60] D.P. Laureano, V. Kirjner, L.C. Ferraro, C.G. Carvalho, J.C.L. Leite, T.P. Hemesath, E.C. Costa, G. Guaragna-Filho, and S. Leistner, Gly183Ser homozygous mutation of the steroid 5-a reductase type 2 (SRD5A2) gene in a Brazilian patient: case report. Journal of pediatric endocrinology & metabolism : JPEM 37 (2024) 1091-1095.

[61] K. Matsubara, H. Iwamoto, A. Yoshida, and T. Ogata, Semen analysis and successful paternity by intracytoplasmic sperm injection in a man with steroid 5α-reductase-2 deficiency. Fertility and sterility 94 (2010) 2770.e7-10.

[62] G.H. Sinnecker, O. Hiort, L. Dibbelt, N. Albers, H.G. Dörr, H. Hauss, U. Heinrich, M. Hemminghaus, W. Hoepffner, M. Holder, D. Schnabel, and K. Kruse, Phenotypic classification of male pseudohermaphroditism due to steroid 5 alpha-reductase 2 deficiency. American journal of medical genetics 63 (1996) 223-30.

[63] F. Vilchis, J.P. Méndez, P. Canto, E. Lieberman, and B. Chávez, Identification of missense mutations in the SRD5A2 gene from patients with steroid 5alpha-reductase 2 deficiency. Clinical endocrinology 52 (2000) 383-7.

[64] P. Mehta, and S. Rajender, Exome sequencing identified compound heterozygous mutations in the SRD5A2 gene in a case of 46,XY ambiguous genitalia. Andrologia 53 (2021) e13937.

[65] H. Zhu, W. Liu, B. Han, M. Fan, S. Zhao, H. Wang, Y. Lu, C. Pan, F. Chen, M. Chen, H. Song, K. Cheng, and J. Qiao, Phenotypic and molecular characteristics in eleven Chinese patients with 5α-reductase Type 2 deficiency. Clinical endocrinology 81 (2014) 711-20.

[66] L. Maimoun, P. Philibert, B. Cammas, F. Audran, P. Bouchard, P. Fenichel, M. Cartigny, C. Pienkowski, M. Polak, N. Skordis, I. Mazen, G. Ocal, M. Berberoglu, R. Reynaud, C. Baumann, S. Cabrol, D. Simon, K. Kayemba-Kay's, M. De Kerdanet, F. Kurtz, B. Leheup, C. Heinrichs, S. Tenoutasse, G. Van Vliet, A. Grüters, M. Eunice, A.C. Ammini, M. Hafez, Z. Hochberg, S. Einaudi, H. Al Mawlawi, C.J. Nuñez, N. Servant, S. Lumbroso, F. Paris, and C. Sultan, Phenotypical, biological, and molecular heterogeneity of 5α-reductase deficiency: an extensive international experience of 55 patients. The Journal of clinical endocrinology and metabolism 96 (2011) 296-307.

[67] Yao CC, Tian RH, Li P, Chen HX, Zhi EL, Huang YH, Zhao LY, Yang C, Zhang L, Li YJ, Li X, Li Z. 2020. Novel compound heterozygous LoF mutations in SRD5A2 may result in disorders of sex development. Zhonghua Yi Xue Za Zhi 100(22) 1699-703.

[68] Vilchis, F., Canto, P., Chávez, B., Ulloa-Aguirre, A., Méndez, J.P., 1997. Molecular analysis of the 5α-steroid reductase type 2 gene in a family with deficiency of the enzyme. Am. J. Med. Genet. 69, 69–72.
